# Long-Term High-Temperature Stress Impacts on Embryo and Seed Development in *Brassica napus*

**DOI:** 10.3389/fpls.2022.844292

**Published:** 2022-04-22

**Authors:** Kateřina Mácová, Unnikannan Prabhullachandran, Marie Štefková, Ioannis Spyroglou, Aleš Pěnčík, Lenka Endlová, Ondřej Novák, Hélène S. Robert

**Affiliations:** ^1^National Centre for Biomolecular Research, Faculty of Science, Masaryk University, Brno, Czechia; ^2^Hormonal Crosstalk in Plant Development, Mendel Center for Plant Genomics and Proteomics, CEITEC MU—Central European Institute of Technology, Masaryk University, Brno, Czechia; ^3^Plant Sciences Core Facility, Mendel Center for Plant Genomics and Proteomics, CEITEC MU—Central European Institute of Technology, Masaryk University, Brno, Czechia; ^4^Laboratory of Growth Regulators, Faculty of Science, Palacký University and Institute of Experimental Botany, The Czech Academy of Sciences, Olomouc, Czechia; ^5^Research Institute of Oilseed Crops, Opava, Czechia

**Keywords:** *Brassica napus*, embryo development, high temperatures, hormonal profiling, oil content, seed development, thermomorphogenesis

## Abstract

*Brassica napus* (rapeseed) is the second most important oilseed crop worldwide. Global rise in average ambient temperature and extreme weather severely impact rapeseed seed yield. However, fewer research explained the phenotype changes caused by moderate-to-high temperatures in rapeseed. To investigate these events, we determined the long-term response of three spring cultivars to different temperature regimes (21/18°C, 28/18°C, and 34/18°C) mimicking natural temperature variations. The analysis focused on the plant appearance, seed yield, quality and viability, and embryo development. Our microscopic observations suggest that embryonic development is accelerated and defective in high temperatures. Reduced viable seed yield at warm ambient temperature is due to a reduced fertilization rate, increased abortion rate, defective embryonic development, and pre-harvest sprouting. Reduced auxin levels in young seeds and low ABA and auxin levels in mature seeds may cause embryo pattern defects and reduced seed dormancy, respectively. Glucosinolates and oil composition measurements suggest reduced seed quality. These identified cues help understand seed thermomorphogenesis and pave the way to developing thermoresilient rapeseed.

## Introduction

Global climate changes with outbreaks of extreme weather harm reproductive development and decrease agronomic crops’ overall yield. Severe weather incidents profoundly affected crop harvest worldwide due to drought and heat during 1964–2007 (Lesk et al., 2016). Affected harvest has been reported in rice (Peng et al., 2004; Guo et al., 2019), maize (Liu et al., 2020), wheat (Liu et al., 2016), soybean (Zhao et al., 2017), tomato (Singh et al., 2017), and cotton (Singh et al., 2007). Predicted increase in temperatures in the future will deepen problems with crop yield (Zhao et al., 2017), which encourages scientific efforts in producing thermotolerant (or stress-tolerant) cultivars. Heat stress (HS) is considered a growing condition above the critical threshold temperature. For most temperate crops (some cereals such as wheat, fruit crops, horticultural crops), the critical temperature is around 30°C (Luo, 2011).

Rapeseed is the second most important crop for oil production with high nutritive quality, currently cultivated on 37.58 million ha with an annual production of 75 million tons and average productivity of 1.99 tonnes/ha (Food and Agriculture Organization of the United Nations, 2020). Total oil production is dependent on several crucial steps leading to plant reproduction and seed production. Seed production depends on (i) the transition to the flowering stage, (ii) flower, ovules and pollen formation, (iii) pollination and fertilization processes (ovule viability, pollen germination, and pollen tube growth), and (iv) embryo, endosperm and seed development, and seed maturation. The flowering stage is the most temperature-sensitive phase of development (Gan et al., 2004). During flowering, the critical temperature for rapeseed plants ranges from 25°C to 32°C depending on the studied cultivars (Polowick and Sawhney, 1988; Morrison et al., 1989; Angadi et al., 2000). The intra- and interspecific genetic variability of the cultivars in their stress response foresees chances for breeding programs (Chen et al., 2019, 2020a).

Specific effects of HS on individual generative growth events have been reported in some agronomically important crops. HS reduces pollen viability and fertilization events in pea (Jiang et al., 2019), rice (Das et al., 2014), and chickpea (Devasirvatham et al., 2013). HS decreased seed set, seed filling, and quality in wheat (Hays et al., 2007; Talukder et al., 2014), rice (Lin et al., 2010), chickpea (Jumrani and Bhatia, 2014), and sorghum (Impa et al., 2019). In *Brassica napus*, several reproduction steps were evaluated for sensitivity to HS. After short heat treatments, rapeseed pollen manifests a limited HS sensitivity (Young et al., 2004; Chen et al., 2020a). Reduced seed weight and seed set were reported in different cultivars (Morrison and Stewart, 2002; Koscielny et al., 2018; Chen et al., 2020a). High-temperature treatments induce changes in seed oil content and photosynthetic activity. For example, the damage to PSII and inhibition of a fatty acid biosynthesis pathway controlled by the transcription factor BnWRI1 might be the major causes of the decreased oil content of *B. napus* seeds that develop under HS (Huang et al., 2019).

The impact of long-term elevated temperatures above critical temperature threshold during the whole reproductive phase was performed on *B. napus* cultivar (N99-508) at 29°C (Elferjani and Soolanayakanahally, 2018), with a more severe outcome than short treatment for seed yield. However, no detailed analysis was so far performed to dissect the causes of this reduced production of viable seeds. Moreover, in the cultivar Aviso, HS altered seed quality and induced pre-harvest sprouting (PHS), correlating with decreased abscisic acid (ABA) levels in mature seeds (Brunel-Muguet et al., 2015). Given that stress resistance is variable in different cultivars, the question remains whether PHS in response to HS is a cultivar-specific trait. PHS is marked by reduced or absent dormancy and activation of processes linked to seed germination. These include sugar metabolism activation, for which the FRUCTOSE 1,6-BIPHOSPHATE ALDOLASE 6 (FBA6) enzyme may be a marker. DORMANCY/AUXIN ASSOCIATED FAMILY PROTEIN 1 (DRM1) and DRM2 proteins are also often used as markers for dormancy release due to their expression in the dormant stages of meristematic tissues, including during seed dormancy (Rae et al., 2014).

Plants adapt their growth and the timing of their development in response to HS by altering homeostasis, transport, and signaling of phytohormones, including auxin and ABA (Vu et al., 2019). Elucidation of hormonal crosstalk and regulation of morphogenesis under HS (thermomorphogenesis) is still in progress in many different crops and tissues (Stavang et al., 2009; Torres et al., 2017). ABA controls various processes in plants, including maintenance of seed dormancy (Seo et al., 2006). ABA is responsible for stress tolerance and acts as a regulator for response to various stress, including HS (Tuteja, 2007; Vishwakarma et al., 2017). ABA increases the production of the chaperone heat shock proteins (HSPs) (and H_2_O_2_) in maize (Hu et al., 2010), wheat (Hu et al., 2018), rice (Zhang et al., 2014), and cucumber (Li et al., 2014). Auxin is involved in the thermomorphogenesis response in seedlings (Vu et al., 2019). Auxin is an essential hormone for embryonic patterning at the early stage of seed development (Verma et al., 2021). Auxin signaling targets components of the ABA signaling pathway to stimulate seed dormancy during seed maturation development (Liu et al., 2013). In addition, auxin biosynthesis through tryptophan is connected to indole glucosinolates, specific secondary metabolites of the *Brassicaceae* family (Eom et al., 2018; Salehin et al., 2019) and secondary seed dormancy (Liu et al., 2019). In Arabidopsis, glucosinolates may trigger thermotolerance by inducing HSP and H_2_O_2_ production (Ludwig-Müller et al., 2000).

A thermosensing pathway, overlapping with components of the circadian clock control, integrates environmental signals, thermal stresses, and phytohormones. Genes related to circadian clocks encode transcription factors arranged in several feedback loops to synchronize plant physiology with the daily changing environment (Sanchez et al., 2011). Circadian gene expression was altered by both cold treatment (Bieniawska et al., 2008) and heat treatment in Arabidopsis and crops (Blair et al., 2019; Li et al., 2019). The photoreceptor PHYTOCHROME A (PHYA) and EARLY FLOWERING 4 (ELF4), a repressive component of the Evening Complex, control induction of flowering, regulate circadian clock loops and take part in the thermosensory mechanism (McWatters et al., 2007; Box et al., 2015; Seaton et al., 2018). Both are temperature-responsive genes (Song et al., 2018; Chen et al., 2020b). One of the repressed targets of the Evening Complex is the thermomorphogenesis-promoting transcriptional factor Phytochrome-Interacting Factor 4 (PIF4; Box et al., 2015). High temperatures reduce the DNA-binding strength of the Evening Complex on the PIF4 promoter, connecting thermosensing with the circadian clock regulation (Ezer et al., 2017). In turn, PIF4 may activate the expression of the florigen FLOWERING LOCUS T (FT) to accelerate flowering in response to warm ambient temperature (Kumar et al., 2012). Furthermore, alternative splicing plays a crucial role in stress response, circadian clocks regulation, and gametophytic development (Sugliani et al., 2010; James et al., 2012; Filichkin et al., 2015; Kulichová et al., 2020; Slane et al., 2020; John et al., 2021). Both high temperatures and altered ABA levels in seeds may regulate the levels of the spliceosome components (Cruz et al., 2014; Jiang et al., 2017).

Even though previous work pointed out the HS effects on viable seed yield, the causes of defective seed production were not investigated. Moreover, most of the studies performed short- to mid-term stress treatment (heat shock), while we were interested in dissecting seed thermomorphogenesis, in other words, how seeds develop while faced to warm ambient temperatures. This study investigated the effects of long-term high-temperature regimes mimicking natural daily rhythm on three *B. napus* cultivars focusing on reproductive growth, especially changes in early seed and embryo development, and seed yield and quality. We measured yield traits and supplemented these macroscopic data with a detailed microscopic analysis of seed and embryo development to examine embryo defects and analysis of seed germination and seedlings (Supplementary Figure S1). We performed hormonal and seed composition profiling and identified development- and hormone-related genes, which could explain the plant HS response in specific development steps and outline possible targets for yield improvement under HS.

## Materials and Methods

### Plant Materials and Growing Conditions

Three *B. napus* spring cultivars, namely Westar, Topas, and DH12075, were used for this study. Seeds were sterilized with 20% bleach, washed twice in sterile distilled water, vernalized at 4°C for 24 h, germinated on plates containing MS medium for 5 days (21°C, 16 h light/8 h dark period, 150 μmol/m^2^/s), and transferred to soil in 0.7 L pots. After 2 weeks in phytotron (21°C, 16/8 L/D period, 150 μmol/m^2^/s), the plants were fertilized with KRISTALON™ Start [N-P-K (19-6-20) + 3% Mg + 7.5% S] and 1 week later transferred to 1.5 L pots. With the first visible flowering buds, the pots were transferred to the greenhouse chambers (Photon Systems Instruments, s.r.o.). Ten plants per each cultivar per replicate in four biological replicates were tested for each temperature regime. Plants from each cultivar were cultivated in blocks of individual pots distant from the plants of other cultivars in each of the greenhouse chambers (120 m^3^ cultivation space) to avoid cross-pollination. The chambers were maintained with a long-day regime (16 h light/8 h dark), LED lights with a light intensity of 150 μmol/m^2^/s, 35%–45% humidity, and 18°C during the night. During the day, control (CT), mid- (MT) and high-temperature (HT) chambers were set to 21°C, 28°C, and 34°C, respectively, with ramping of the temperature up and down by 4°C per hour (Supplementary Figure S1). During this study, the temperature growth regime 21/18°C is defined as the optimal growth temperature (control temperature, CT), 28/18°C is a suboptimal growth temperature (mid-temperature, MT), and 34/18°C is a stressful high growth temperature (high-temperature, HT). The temperature was uniformly distributed in each chamber by an air circulation system. Temperatures were controlled with a thermostat in the middle of each chamber, situated on the wall at 150 cm from the ground, and adjusted by water heaters and an adiabatic cooling system. Plants were well watered manually in the trays to avoid any effect associated with drought stress. Plants were once fertilized with KRISTALON™ Fruit and Flower [N-P-K (15-5-30) + 3% Mg + 5% S] at the flowering start and kept in the respective chamber till seed harvest. The experiment was performed between October 2019 and March 2020.

### Plant Phenotyping Measurements

The number of leaves (NL) was counted at the beginning of flowering as a controlling trait. The length of the main flowering stem (LMS, cm), the duration of flowering time (FT, days), the number of primary branches on the main stem (NB), the number of flowers on the main stem (NF), and the number of ovules per pistil were evaluated during each experiment (four replicates, 10 plants per cultivar). FT was measured as the duration between the first and the last opened flowers.

Pollen viability was tested by both acetocarmine and Alexander staining. Alexander staining solution was prepared according to Alexander (1969). Pollen grains were tapped on a microscopic slide with 10 μl of Alexander solution, incubated at 50°C for 1 h, and counted under the light microscope Zeiss Axioscope.A1. For acetocarmine solution, 2% carmine solution was made in 95% glacial acetic acid (Marutani et al., 1993). Pollen grains were stained for 10 min and evaluated using the same microscope.

For silique length (SL, cm), 30 flowers were hand-pollinated for each cultivar (five flowers on six plants, four biological replicates). The siliques were measured every 24 h for 11 days to monitor silique growth over time. For correlation analysis, the final length and number of seeds (NS) per silique were recorded at harvest. Siliques with a length <1 cm were excluded (no seeds). The Relative Growth Rate (RGR) from the exponential growth model was used to examine the temperature effect on silique growth after pollination. RGR is estimated by the coefficient (*r*) from the exponential function: $Y=C*e^{rt}$ , where *Y* is the variable modeled and *C*, a constant term. When *r* = 0.1, the silique grows by an average of 10% every time point *t*.

### Seed Phenotyping

Seeds from five hand-pollinated flowers on 10 plants were evaluated for the number of seeds and different phenotypes of mature seeds after harvest. Moreover, seeds from two plants were collected at 3, 4, 5, 6, 7, and 8 DAP (days after pollination). The ovule number (NO) was calculated by adding the number of developed seeds and unfertilized ovules, counted under the stereomicroscope in all siliques. From siliques at 5–8 DAP, the number of fertilized, aborted, and non-fertilized was similarly evaluated. Seeds were cleared in a chloral hydrate solution (chloral hydrate, Sigma C8383/Glycerol/Water, 8/1/3 w/v/v) for embryo phenotyping at DAP indicated in the text. Observations were made using a light microscope Zeiss Axioscope.A1 equipped with DIC optics and ZEN blue software for picture analysis at CEITEC MU Cell Imaging Core Facility. Premature seed germination (pre-harvest sprouting, PHS) was evaluated at 26 DAP. After harvest, seeds were stored in paper bags at room temperature. The 100-seed weight and germination rate with seedling viability assessment were analyzed 4 months after harvest to allow equal seed desiccation, stored in paper bags in the dark at room temperature.

**Table 1:** Evaluation under the stereomicroscope of the number of ovules, fertilized and unfertilized seeds per silique.

	Conditions	NO^*^	No. ovules	No. siliques	Seeds (%)	Non-fertilized (%)	Aborted (%)	No. seeds
DH12075	CT	24.78–27.66	1,590	61	90.81	8.05	1.14	1,752
	MT	24.29–27.39	2,054	80	78.06	19.45	2.50	1,445
	HT	26.05–28.79	3,140	114	47.27	51.81	0.93	1,408
Topas	CT	21.33–24.05	1,794	79	89.25	7.41	3.34	1,254
	MT	21.98–24.53	1,951	84	79.09	14.30	6.61	1,422
	HT	22.65–25.03	2,775	118	38.58	46.12	15.30	1,889
Westar	CT	26.31–33.45	2,559	85	92.13	6.84	1.03	1,841
	MT	24.78–31.19	2,412	86	69.59	20.95	9.46	1,777
	HT	23.57–29.96	2,386	99	32.55	55.05	12.40	1,738

* NO: Number of ovule per silique presented as 95% CI.

### Data Analysis

The results (multiple pairwise comparisons between different conditions-temperatures) were analyzed using generalized linear mixed models (Poisson or negative binomial mixed model for count data and linear mixed model for continuous data), and ANOVA followed by Tukey’s *post hoc* test in R software (R Core Team, 2014) in RStudio (RStudio Team, 2019). Mixed models were used, so the randomness between the different batches is considered (McCulloch and Neuhaus, 2005). The level of statistical significance was set at *p* ≤ 0.05 for all tests. Packages “glmmTMB” and “emmeans” were used for the fitting of mixed models and implementing pairwise comparisons, respectively (R Core Team, 2014; Brooks et al., 2017; RStudio Team, 2019; Russell, 2020).

### Seed Metabolites Content

Harvested mature seeds were analyzed for their oil composition and glucosinolates content. Three technical replicates for each of the three biological replicates from CT and MT regimes were analyzed for the three cultivars. Mature seeds were collected for each plant separately. A total of 210 seeds from three plants (3× 70 seeds, more than 0.5 g) was sampled for one technical replicate. Seeds for each technical replicate originated from three different plants from the same set of plants grown for phenotyping. Each biological replicate for seed metabolites content analysis corresponds to plants grown from a biological replicate for the phenotyping analysis. Sample measurements were performed by a spectrophotometer FT-NIR Antaris II (Thermo Fisher Scientific Inc., United States) on the integration sphere in reflectance mode in a spectral range of 10,000–4,000 cm^−1^ using OMNIC for Antaris software. Whole seeds were measured in rotary circular cuvettes with quartz bottom permeable for NIR radiation. The resulting spectrum of each sample was obtained as an average of 64 scans with a resolution of 2 cm^−1^. Calibration models for quantitative analysis of the oil, main fatty acids (palmitic, stearic, oleic, linoleic, linolenic), and glucosinolates content were developed using Partial Least Squares algorithm in Thermo Scientific TQ Analyst software. Data measured by routine laboratory reference methods were used to construct FT-NIR calibration models. Determination of oil content was performed by extraction method according to ISO 659 (2009), which defines the weight determination of oil content after extraction with petroleum ether, distillation of the solvent, and drying the extracted fat. The dry matter content was determined by a gravimetric method, according to ISO 665 (2000), after 4 h of drying at 103°C. To determine the GSL content, the HPLC/UV–VIS method, according to ISO 9167-1 (1992), was used, which defines the GSL determination in the form of desulfoglucosinolates. The representation of individual fatty acids in the form of fatty acid methyl esters was detected by GC/FID according to the internal methodology of the Research Institute of Oilseed Crops. All methods used are validated and routinely used. Data of total oil are presented as % content in dry matter. The content of each fatty acid is shown as % of the total oil content. The nitrogen compound content (%) is a measure of protein content in seeds. The number represents the mean value ± SD. Statistical evaluation for significant difference in heat treatments was performed by a paired Student’s *t*-test (*, **, and *** correspond to value of *p* 0.05 > *p* > 0.01, 0.01 > *p* > 0.001, and *p* < 0.001, respectively). The fatty acid composition is also expressed by the ODP (Oleic Desaturation Proportion) and LDP (Linoleic Desaturation Proportion), derived from the formulae: $ODP=100\times \frac{\left(\%\mathrm{Linoleic}+\%\mathrm{Linolenic}\right)}{\left(\%\mathrm{Oleic}+\%\mathrm{Linoleic}+\%\mathrm{Linolenic}\right)}$ and $LDP=100\times \frac{\left(\%\mathrm{Linolenic}\right)}{\left(\%\mathrm{Linoleic}+\%\mathrm{Linolenic}\right)}$ (Cherif et al., 1975). These parameters are proportional to the action of enzymes for the desaturation of oleic and linoleic acids, respectively. Graphs are done with GraphPad Prism 9.0.

### Hormonal Measurements

IAA, its metabolites, and ABA were determined following the methods described by Pěnčík et al. (2018). Samples of Westar pistils, 5 DAP, and 26 DAP seeds (two biological replicates, each with three technical replicates) containing 10 mg of frozen homogenized tissue were extracted with 1 ml of 50 mM phosphate buffer (pH 7.0) containing 0.1% sodium diethyldithiocarbamate and a mixture of stable isotope-labeled internal standards. One portion of the extract (200 μl) was acidified with HCl to pH 2.7 and purified by in-tip micro solid-phase extraction (in-tip μSPE). Another 200 μl of the extract was purified directly without acidification to determine IAOx. The last 200 μl portion was derivatized by cysteamine, acidified with HCl to pH 2.7, and purified using in-tip μSPE to determine IPyA. After evaporation under reduced pressure, samples were analyzed using HPLC system 1,260 Infinity II (Agilent Technologies, United States) equipped with Kinetex C18 column (50 mm x 2.1 mm, 1.7 μm; Phenomenex) and linked to 6,495 Triple Quad detector (Agilent Technologies, United States). Data are presented as pmol for 1 g of fresh tissue. The number represents the mean value ± SD. Statistical evaluation for significant difference in heat treatments was performed by a paired Student’s *t*-test (*, **, and *** correspond to value of *p* 0.05 > *p* > 0.01, 0.01 > *p* > 0.001, and *p* < 0.001, respectively).

### RNA Extraction and RT-qPCR

Tissue (50–100 mg per replicate) collected from CT and HT regimes was directly frozen and stored in a −80°C freezer. For isolation, samples were ground into a fine powder using mortar or ceramic beads added to 2 ml Eppendorf tubes. The total RNA from leaves, pistils, and young seeds at the early globular embryonic developmental stage (7 DAP from CT and 5 DAP from HT) were isolated following a protocol for Trizol (Invitrogen). RNA from 26 DAP seeds was isolated using a NucleoSpin RNA Plant and Fungi kit (Macherey-Nagel) according to the manufacturer’s protocol. All samples were treated with a rDNase, RNase-free (Macherey-Nagel). M-MLV Reverse Transcriptase (Promega) was used for reverse transcription on two μg of DNA-free RNA.

Expression of genes involved in auxin and ABA biosynthesis, degradation and signaling, and genes related to mRNA splicing pathway and regulators of flowering and thermogenesis were quantified. Gene sequences for primer design were available in the Genbank database (LOC number listed in Supplementary Table S1). The selection of the candidate reference genes for normalization was made using the combination of the algorithms provided by Normfinder (Andersen et al., 2004), geNorm (Vandesompele et al., 2002), and BestKeeper (Pfaffl et al., 2004). The geometric mean of the Cq values of the selected genes was used as a normalization factor for calculating the log2-fold change. The selected reference genes were *BnaACT7* (leaves, pistils, and 26 DAP seeds), *BnaEIF5A* (leaves, pistils, young seeds, and 26 DAP seeds), and *BnaTMA7* (pistils and young seeds). These genes were chosen as their expression is not affected by the temperature treatment in the given tissues. Temperature-dependent expression of targeted genes (Supplementary Table S1) was evaluated in the presented tissues (leaves, pistils, seeds). All primers sequences used in this study are listed in Supplementary Table S1. Gene transcript abundance was quantified by RT-qPCR using FastStart Essential DNA Green Master (Roche) on a Lightcycler 96 (Roche). The experiment was performed with three biological replicates, each with three technical replicates. Analysis of fold changes was performed using the 2^−ΔΔCt^ method (Livak and Schmittgen, 2001). The significance of the expression changes was evaluated by a *t*-test. The qPCR reactions were performed according to MIQE Guidelines (Bustin et al., 2009).

## Results

### High-Temperature Treatment Altered the Overall Plant Growth and Architecture

**Figure 1:**
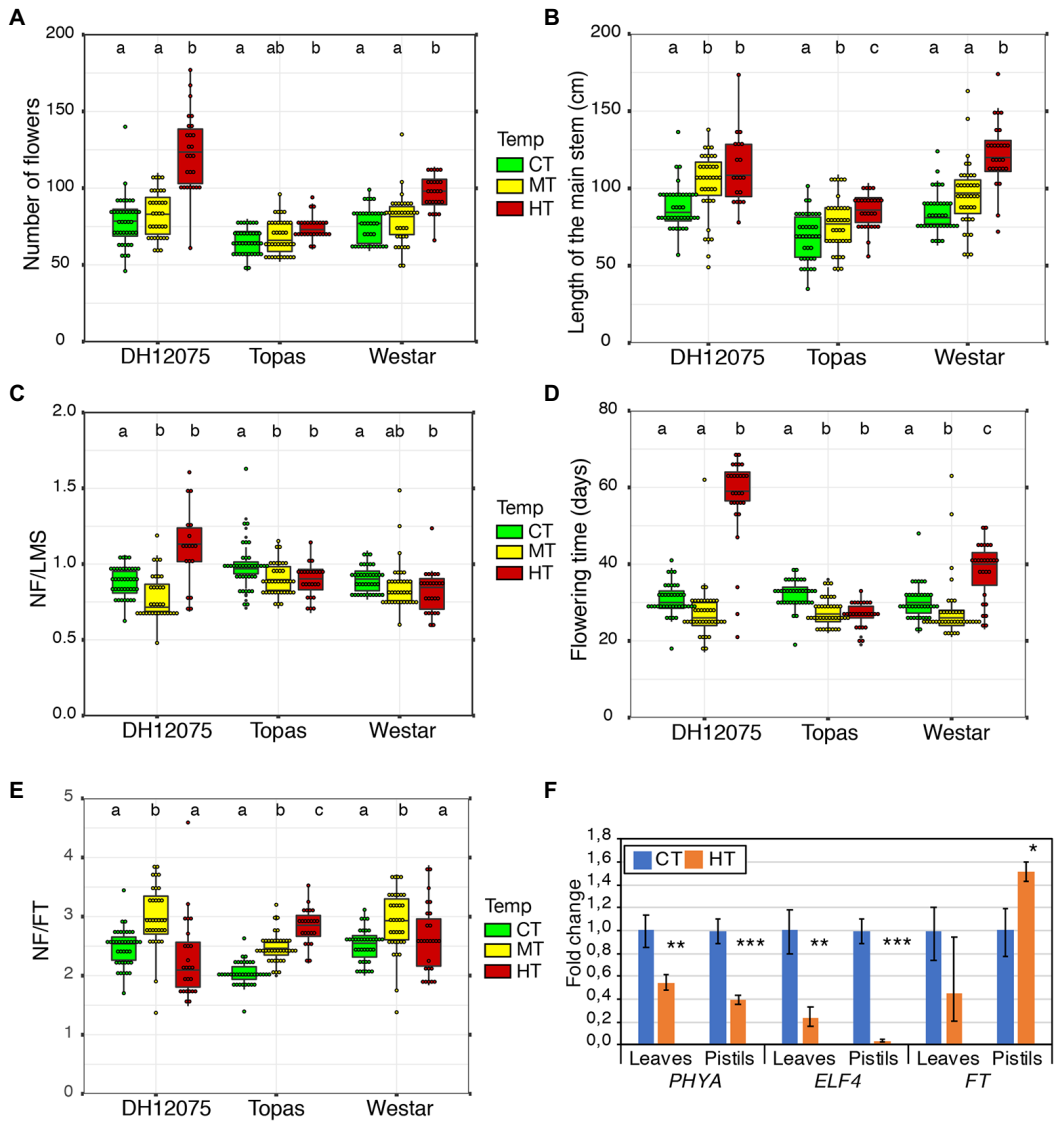
Effects of high temperatures on flowering traits and seed production. **(A–E)** The number of flowers (NF; **A**), the length of the main stem (LMS; **B**), the number of flowers per cm of inflorescence (NF/LMS; **C**), the flowering time (FT; **D**), and the number of flowers per day (NF/FT; **E**) were quantified in DH12075, Topas, and Westar cultivars at CT (green), MT (yellow) and HT (red). The data are presented as boxplots. The box represents the interquartile range, and the line inside the box represents the median. Each dot represents a measurement. The Pearson correlation coefficient between LSM, FT, and NF, is shown in Supplementary Table S2. Boxes with the same letters (a, b, and c) within each cultivar do not differ significantly (*p* < 0.05). **(F)** Expression analysis by RT-qPCR of *BnaPHYA*, *BnaELF4*, and *BnaFT* in Westar leaves and pistils. The graph displays the fold changes in expression between CT (blue) and HT (orange). Asterisks indicate statistically significant difference in HT in a paired Student’s *t*-test (*t*-test; *, **, and *** correspond to value of *p* 0.05 > *p* > 0.01, 0.01 > *p* > 0.001, and *p* < 0.001, respectively). Primers and LOC information are presented in Supplementary Table S1.

Three *B. napus* cultivars were evaluated for main growth characteristics in the three temperature regimes. At the beginning of the experiment, NL (number of leaves) was evaluated with no significant difference among the temperatures demonstrating a comparable vegetative growth of all the plants randomly distributed to the different greenhouse chambers (Supplementary Figure S2A). For all the cultivars, significantly higher LMS (length of the main stem) and NF (number of flowers) were detected in HT conditions, while MT only increases LMS to a limited extend and cause no change of NF (Figures 1A,B; Supplementary Figure S2). The flowering stem’s growth speed is more accelerated than the production of flowers by the floral meristem on the main stem, resulting in a decreasing trend in NF/cm ratio (NF/LMS; Figure 1C). A strong correlation was detected in CT (control growth temperatures, 21/18°C) between LMS and NF with a comparable level among all three cultivars (>0.77). In MT (mid growth temperature, 28/18°C), a strong correlation was maintained in Topas (0.90) and Westar (0.74) but dropped to 0.43 in DH12075. In HT (high growth temperature, 34/18°C), on the contrary, this correlation dropped for Topas (0.44) to almost no correlation for Westar (0.22) and increased to 0.77 for DH12075 (Supplementary Table S2).

Differential growth imposed by MT and HT was also reflected in flowering time (FT). In MT, Westar and Topas reacted with shortening their flowering time (Figure 1D; Supplementary Figure S2). Topas kept a reduced flowering time in the HT regime due to problems with the floral meristem, restricting growth, while DH12075 and Westar displayed a significantly longer flowering time in HT (Figure 1D; Supplementary Figure S2). Therefore, these two cultivars developed more flowers per day in MT but not HT (Figure 1E). On the other hand, due to a shorter flowering time, Topas had a significantly higher number of flowers per day in both MT and HT (Figure 1E). In general, a strong correlation was found between the flowering time and the number of flowers in CT and MT, with a decreasing tendency to a low correlation in HT (Supplementary Table S2). Moreover, all the cultivars developed more branches in HT treatment (Supplementary Figure S2).

Longer main stems, prolonged flowering duration, and more branches in DH12075 and Westar cultivars (Figure 1; Supplementary Figure S2) suggest changes in hormonal levels, maintenance of growth, and decreased apical dominance in HT growth conditions. On the other hand, in Topas, the floral meristem arrested its growth earlier at HT and, therefore, developed more flowers per day. However, the other measured parameters showed that Topas appeared less negatively affected by both MT and HT growth regimes on the whole plant level than the other cultivars (Supplementary Figure S2D).

The relative expression of *PHYA* and *ELF4*, genes related to thermosensing, was assessed. We found both *PHYA* and *ELF4* to be significantly downregulated in HT in leaves and pistils (Figure 1F). The expression of the *FLOWERING LOCUS T* gene, indirectly regulated by ELF4 through the Evening Complex in response to high temperature (Kumar et al., 2012; Box et al., 2015; Ezer et al., 2017), was not significantly changed in leaves, but significantly upregulated in pistils (Figure 1F). A lower amount of *PHYA* and *ELF4* may de-repress the thermomorphogenesis pathway (Ezer et al., 2017).

### High Temperature Decreases the Ovule Fertilization Rate but Does Not Affect the Viability of Pollen Grains

The success of reproduction relies on the regulated development of ovules within the pistil and pollen grains inside the anthers. It is also determined by the proper timing of pollination and fertilization events for subsequent seed development. The number of ovules per pistil (NO) was comparable in all the temperature regimes (Table 1; Supplementary Figure S3A). For Westar, we found a minimal but significant difference between CT and HT, which may be caused by the higher variability of this trait in the Westar cultivar, but its biological relevance is relatively small.

Pollen viability decreased by less than 5% by any temperature treatments (MT and HT), which was not considered a significant effect (Supplementary Figures S3B**–** D). Fertilization and abortion rates were monitored in siliques from 5 to 8 DAP. In all three cultivars, the percentage of non-fertilized ovules increases with increasing temperatures, resulting in fewer developing seeds. Moreover, Topas and Westar cultivars also exhibited an increasing percentage of aborted seeds at the early stages of development. On the contrary, the abortion rate in DH12075 decreased in HT compared to CT and MT (Table 1).

**Figure 2:**
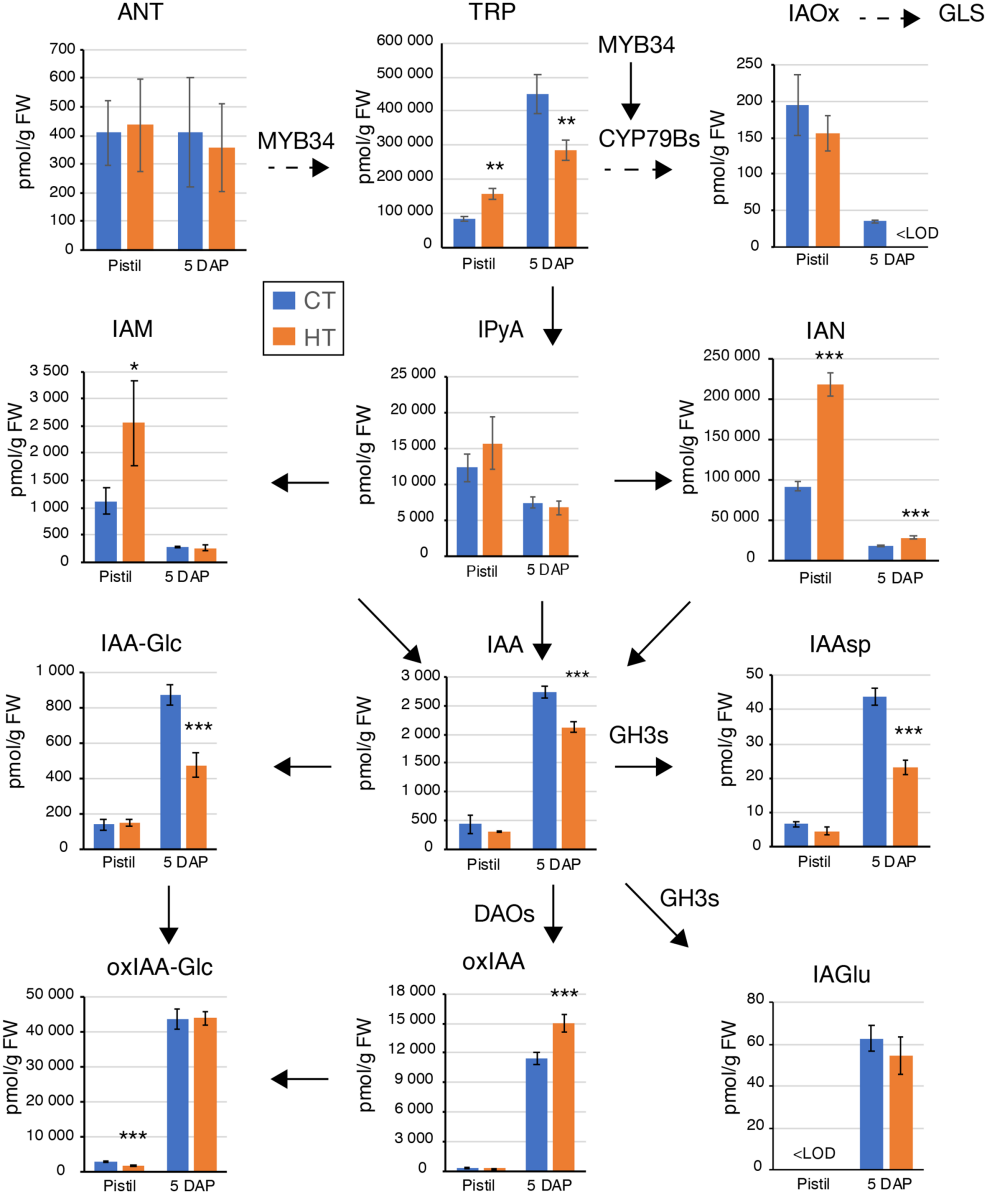
Differences in auxin and auxin metabolites levels induced by high temperatures in Westar pistils and 5 days after pollination (DAP) seeds. Graphs displaying the levels (pmol/g FW) of anthranilate (ANT), tryptophan (TRP), indole-3-acetaldoxime (IAOx), indole-3-pyruvic acid (IPyA), indole-3-acetamide (IAM), indole-3-acetonitrile (IAN), indole-3-acetic acid (IAA), IAA-glucose (IAA-Glc), IAA-aspartate (IAAsp), IAA-glutamate (IAGlu), 2-oxoindole-3-acetic acid (oxIAA), and oxIAA-glucose (oxIAA-Glc). Measurements were done in two biological replicates, five technical replicates in Westar pistils and 5 DAP seeds from plants grown at CT (blue) and HT (orange). Shown is the average ± SD of one of the two biological replicates. <LOD, below the limit of detection. Asterisks indicate statistically significant difference in HT in a paired Student’s *t*-test (*t*-test; *, **, and *** correspond to value of *p* 0.05 > *p* > 0.01, 0.01 > *p* > 0.001, and *p* < 0.001, respectively). Arrows indicate the direction of the biosynthesis pathway (plain arrow, direct reaction; dashed arrow, multiple-step enzymatic reactions). The names closed to the arrows indicate the enzymes involved in the reactions and whose gene expression was tested in Figure 3. Source data are shown in Supplementary Table S3.

**Figure 3:**
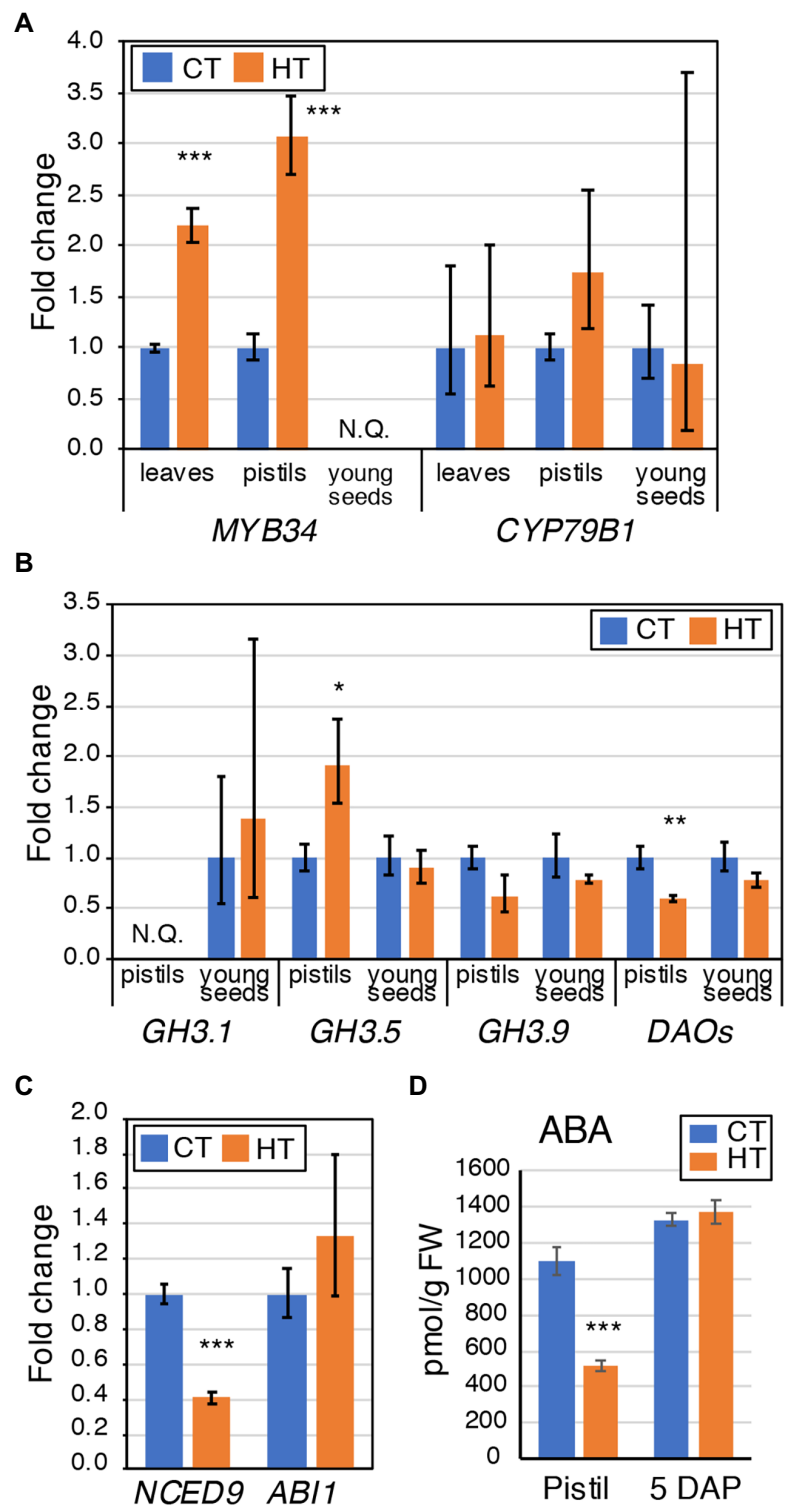
Expression analysis of genes involved in auxin homeostasis and ABA levels in Westar leaves, pistils, and young seeds. **(A)** Expression analysis by RT-qPCR of *BnaMYB4* and *BnaCYP79Bs* in Westar leaves (blue), pistils (orange), and young seeds (grey). **(B)** Expression analysis by RT-qPCR of *BnaGH3.1* (blue), *BnaGH3.5* (orange), *BnaGH3.9* (grey), and *BnaDAOs* (yellow) in Westar leaves, pistils, and young seeds. NQ, not quantified. **(C)** Expression analysis by RT-qPCR of *BnaNCED9* and *BnaABA1* in Westar pistils (blue). **(A–C)** The graphs display the fold changes in expression between CT (blue) and HT (orange). Asterisks indicate statistically significant difference in HT in a paired Student’s *t*-test (*t*-test; *, **, and *** correspond to value of *p* 0.05 > *p* > 0.01, 0.01 > *p* > 0.001, and *p* < 0.001, respectively). Primers and LOC information are presented in Supplementary Table S1. **(D)** Graph showing the ABA levels (in pmol/g FW) in Westar pistils and 5 DAP seeds grown at CT and HT. Shown is the average ± SD. Asterisks indicate a statistically significant difference in HT in a paired Student’s *t*-test (*t*-test; *** corresponds to value of *p* < 0.001). Source data are presented in Supplementary Table S4.

ABA and auxin metabolites content were quantified in pistils from CT and HT in Westar to understand why the fertilization rate was reduced. Increased levels of tryptophan (TRP), indole-3-acetamide (IAM), and indole-3-acetonitrile (IAN) were found in pistils without any changes in indole-3-acetic acid (IAA) and degradation metabolites levels except for decreased levels of 2-oxoindole-3-acetyl-1-O-ß-d-glucose (oxIAA-Glc; Figure 2; Supplementary Table S3). Expression of *MYB34*, encoding an MYB transcription factor regulating the expression of the *CYP79B2/3* enzymes converting Trp into indo-3-acetaldoxime (IAOx; Celenza et al., 2005), was significantly increased in HT pistils and leaves (Figure 3A). However, no significant changes in the expression of *CYP79B2/3* in HT pistils and leaves were observed (Figure 3A). Regarding auxin degradation, only *GRETCHEN HAGEN3.5* (*GH3.5*) is significantly upregulated. GH3 enzymes catalyze the conjugation of IAA with amino acids (Wojtaczka et al., 2022). Despite GH3.5 upregulation in pistils, the Asp-conjugated IAA levels remain unchanged, and GLU-conjugated IAA levels are below detection limits (Figure 2). Also, the *DAO* genes expression was down-regulated in pistils at HT (Figure 3B). DAOs are auxin oxidases involved in auxin degradation by the formation of oxIAA from IAA (Mellor et al., 2016). oxIAA is further metabolized by conjugation with glucose into oxIAA-Glc, which levels were decreased in pistils at HT (Figure 2). We did not observe any phenotypic changes of non-pollinated pistils and the auxin homeostasis seems to be maintained in HT conditions. Therefore, the increased TRP, IAM, and IAN levels may be related to secondary metabolites protection rather than auxin biosynthesis. Also, we found a reduced expression of ABA biosynthetic genes *NCED9* and significantly lower ABA content (Figures 3C,D; Supplementary Table S4). The non-accumulation of ABA in pistils developed at HT may suggest that they are less protected against temperature stress, which may eventually contribute to the reduced fertilization rate.

### High Temperatures Accelerate Seed Development and Alter Embryo Patterning

**Figure 4:**
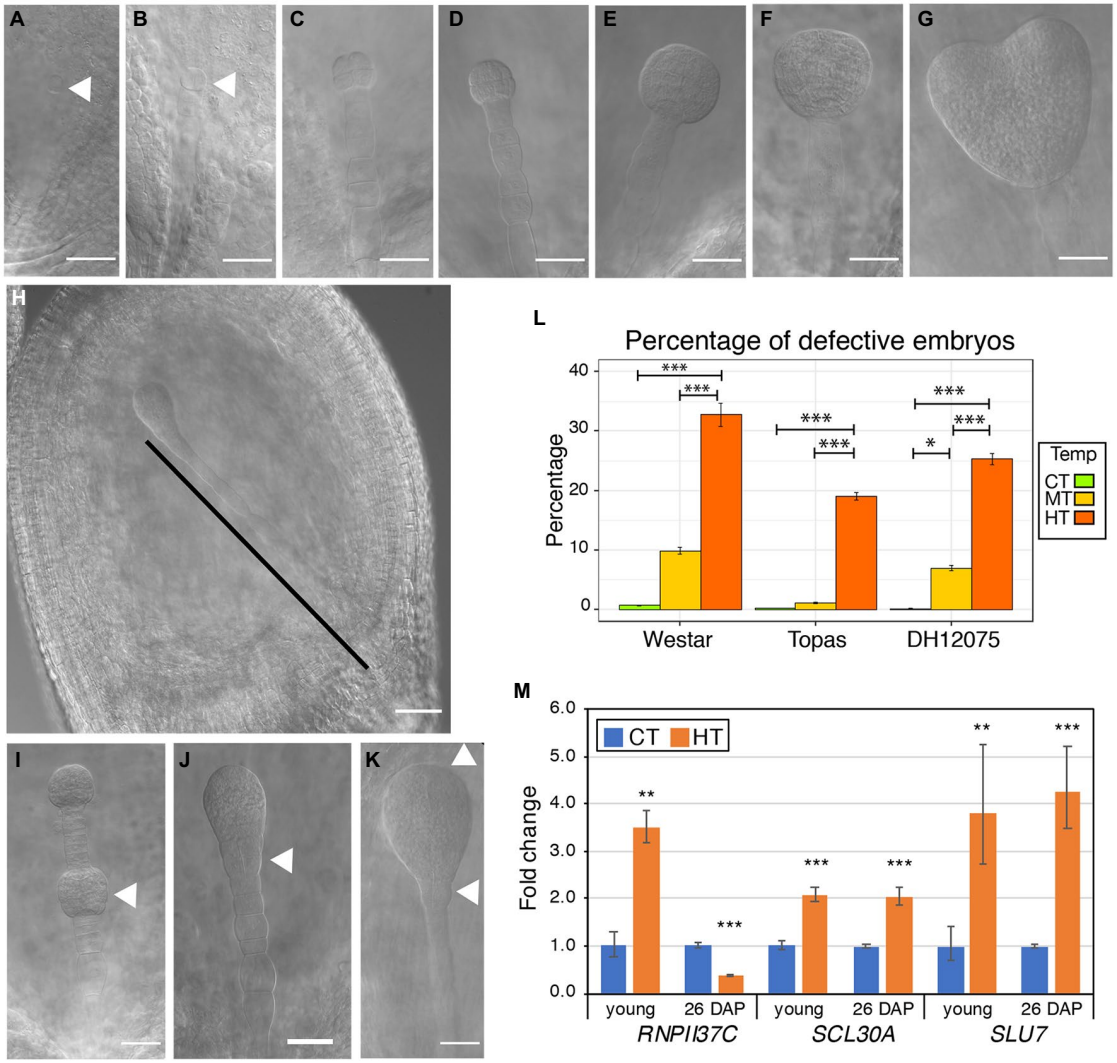
Effects of high temperatures on embryo development. **(A–G)** Representative photos of embryos of *Brassica napus* plants grown at CT. The developmental stages of the embryos are one-cell **(A)**, two-cell **(B)**, 8-cell **(C)**, early globular **(D)**, late globular **(E)**, transition **(F)**, and heart **(G)**. The arrowheads in **(A,B)** indicate the apical cell and proembryo. **(H–K)** Range of defective embryos observed in *B. napus* plants grown at MT and HT between 6 and 8 DAP. **(H)** Seed containing an embryo with supernumerary suspensor cells. The black line marks the suspensor. **(I)** Embryo (7 DAP) with cell proliferation in the suspensor, leading to a secondary embryo (arrowhead). **(J)** Embryo (7 DAP) with cell proliferation in the root pole (arrowhead). **(K)** Embryo (8 DAP) without initiation of cotyledon primordia and with unstructured root pole (arrowheads). Scale bars represent 50 μm **(A–K)**. Uncropped pictures are presented in Supplementary Figure S5. **(L)** Graph displaying the percentage of defective embryos in young seeds of Westar, Topas, and DH12075 plants grown at CT (green), MT (yellow), and HT (orange). Error bars represent the 95% CI. Asterisks indicate a statistically significant difference in MT and HT in an ANOVA followed by Tukey’s *post hoc* test (* and *** correspond to value of *p* 0.05 > *p* > 0.01 and *p* < 0.001, respectively). **(M)** Expression analysis by RT-qPCR of *BnaRNPII37C*, *BnaSCL30A*, and *BnaSLU* in Westar young seeds and 26 DAP seeds. The graph displays the fold changes in expression between CT (blue) and HT (orange). Asterisks indicate statistically significant difference in HT in a paired Student’s *t*-test (*t*-test; ** and *** correspond to value of *p* 0.01 > *p* > 0.001 and *p* < 0.001, respectively). Primers and LOC information are presented in Supplementary Table S1.

Seeds were investigated to monitor embryo development from 3 DAP to 8 DAP (Table 2). The early embryonic development in *B. napus* follows the same dynamics described in Arabidopsis (Verma et al., 2021). Following fertilization, the zygote elongates within the 3 days after pollination (DAP). The zygote divides asymmetrically to give rise to a small apical cell and an elongated basal cell (Figure 4A). At 3 DAP, all the cultivars exhibited embryos in the zygote or one-cell stage. The basal cell will divide anticlinally to form a cell file connecting the apical cell and its progeny to the seed coat cells. The apical cell undergoes three rounds of symmetrical divisions to form an 8-celled embryo (Figures 4B,C). At CT, the three studied cultivars have 8-cell embryos at 5-to-6 DAP in Topas and Westar and 6-to-7 DAP in DH12075 (Table 2). The follow-up cell divisions are asymmetrical with the formation of the embryonic epidermal layer (protoderm) and the specification of the root pole at the lower tier of the embryo to form an early globular embryo between 6 and 8 DAP (Figure 4D). Then, the ground and vascular tissues are specified in the mid-globular stage at 8 DAP (Figure 4E). After 8 DAP, the shoot apex is specified in the upper tier at the late-globular stage, followed by the emergence of two cotyledon primordia from the transition stage (Figure 4F) to form a heart embryo (Figure 4G). The embryo development is accelerated with increasing temperatures, leading to two developmental stages difference at 6 DAP (Table 2; Supplementary Figure S4). Faster embryonic development may affect the synchronous development of the seed coat and the endosperm and eventually lead to a higher abortion rate in later stages (Table 1). Less than 1% of defective embryos were observed in CT for all cultivars. This number significantly increased for DH12075 (6%) and Westar (10%) in MT, and for all the cultivars in HT (DH12075, 25.45%; Topas, 16.2%, and Westar, 39.58%; Figure 4L; Supplementary Figure S4).

**Table 2:** Most abundant embryo stages per DAP (>20% of the embryos per silique at the given DAP).

	Conditions	Embryonic stage						
		Zygote	1-cell	2-cell	8-cell	EG	MG	LG
DH12075	CT	3	3–4	5–6	6–7	7–8	>8	>8
	MT	3	3	4	4–5	5–6	6	7–8
	HT	3	3	4	4–5	5	6	7
Topas	CT	3	3–4	4–5	5–6	6–7	7–8	8
	MT	-	3	4	4–5	5	6	6–7
	HT	3	3	3–4	4	5	5–6	6
Westar	CT	3	3–4	5	5–6	7	8	8
	MT	3	3–4	4	5	6	6	7–8
	HT	-	3	3	4	5	5	6

Six DAP is highlighted to illustrate the shift in the embryo development timing. EG, early-globular; MG, mid-globular; LG, late-globular.

Defective embryonic patterning was observed at 6, 7, and 8 DAP. The defective embryos exhibited several altered cell division patterns in the embryonic lower tier and cell proliferation in the suspensor altering the apical-basal embryonic axis (Figures 4H–K; Supplementary Figure S5). We observed elongated embryos with a proliferating lower-tier domain (Figure 4J). The aberrant divisions in the lower tier domain and the upper suspensor cells ill-defined the root pole and the shoot-root junction (Figures 4J,K). The root pole appears not to be correctly specified (Figures 4J,K). Aberrant or misregulated cell divisions in the apical embryo domain cause incomplete cotyledon development (Figure 4K). The suspensor cells proliferated, resulting in a long cell file (Figure 4H), never observed in CT. In extreme cases, some suspensor cells start dividing periclinally and, after several rounds of division, appears what looks like a secondary embryo (globular shape; Figure 4I).

Because the observed embryonic phenotypes are similar to known mutants with altered auxin homeostasis, we quantified auxin levels in Westar seeds, containing embryos ranging from 8-cell to early globular stages. A decrease in TRP, IAA, and some degradation metabolites (IAAsp and IAA-Glc) and an increase in oxIAA were detected, indicating altered auxin homeostasis in HT (Figure 2). However, the expression of *GH3s*, *CYP79Bs*, and *DAOs* was not significantly changed in seeds bearing early globular embryos in HT (Figures 3A,B). In addition, temperature-induced misregulation of the spliceosome activity may affect embryo development. Therefore, we analyzed whether HT would alter the expression of *SLU7*, *RNPII30C*, and *SCL30A* genes, involved in the alternative splicing pathway. All three were significantly upregulated in the young seeds developed at HT (Figure 4M). Both MT and HT and altered ABA levels in seeds may regulate the levels of the spliceosome components (Cruz et al., 2014; Jiang et al., 2017). But no changes in ABA levels were detected in 5 DAP seeds (Figure 3D; Supplementary Table S4). As one would expect an increase in ABA linked to the applied temperature stress, the steady ABA levels may relate to the abortion of seeds due to lower stress protection (Table 1). Together all these data suggest that the decrease in rapeseed seed yield due to warm growth temperatures is mainly caused by a decreased fertilization rate and defective embryo development. The perturbance of IAA homeostasis and spliceosome activities by warm ambient temperatures may partly be responsible for the embryonic patterning defects.

### Reduced Seed Yield at High Temperatures Is the Result of Seed Abortion and Defective Embryo Development

Silique length (SL) is believed to correlate with the number of seeds they contain (Bac-Molenaar et al., 2015). In HT, in all three cultivars, we have noticed that about 50% of the ovules are not fertilized and that only 32%–47% of the ovules will develop into seeds (Table 1). Therefore, siliques were assessed to their growth rate for 11 days from hand pollination. Siliques from all three cultivars grew on average by 22%–23% daily at CT during the 11 days (Supplementary Figure S6). This growth rate significantly decreased when growth temperature increased to reach a daily growth rate of 18%–20% and 10%–15% at MT and HT, respectively, for all cultivars. The growth rate at HT was, therefore, half the rate than at CT. There is a strong correlation (0.92–0.96 and 0.92–0.99) between the reduced seed number per silique and the reduced growth rate of the siliques at MT and HT, respectively, compared to CT (0.63–0.83; Supplementary Table S6).

**Figure 5:**
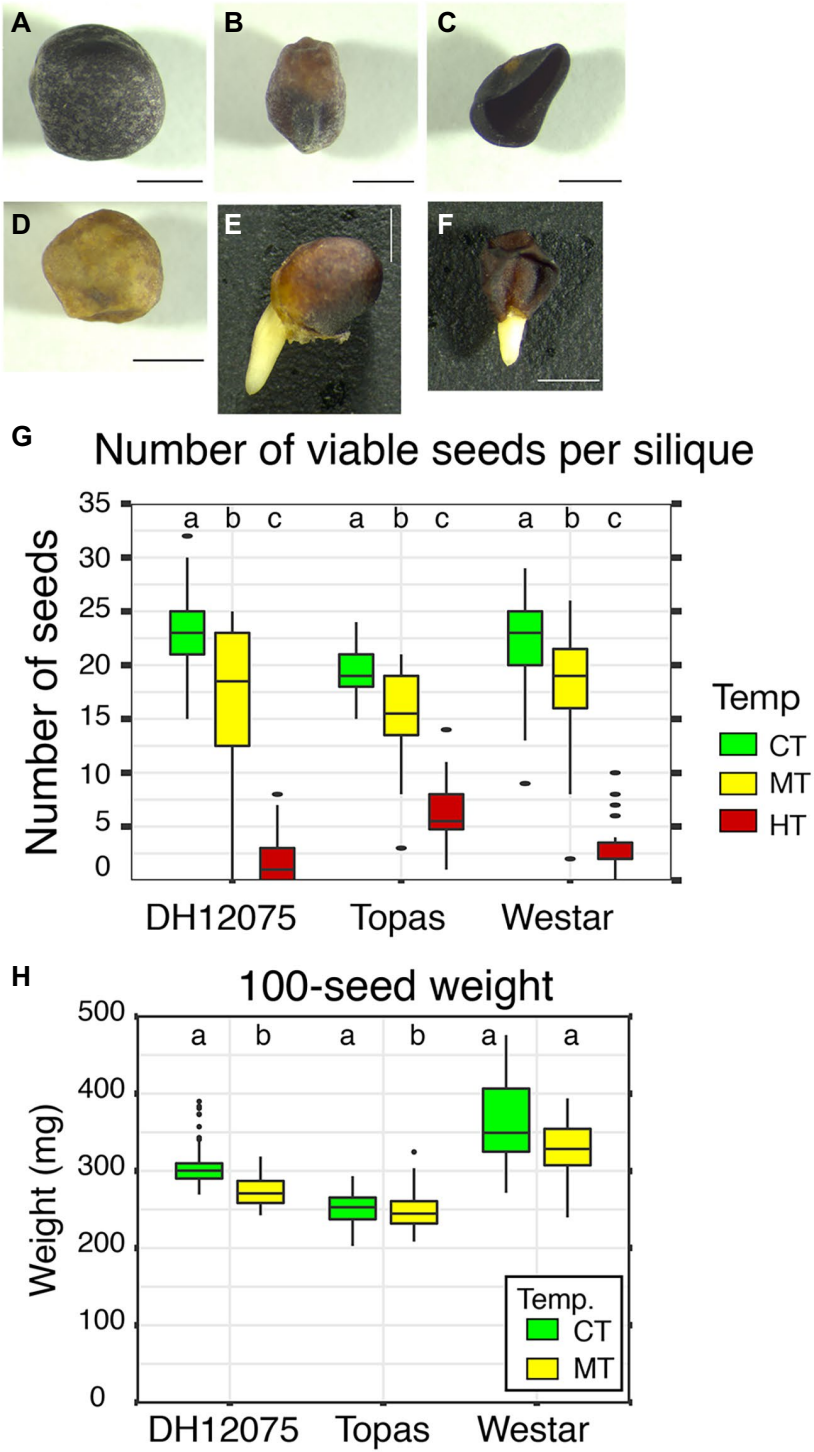
Effects of high temperatures on seed development. **(A–F)** Range of phenotypes observed in mature seeds. We observed normal fully filled seed **(A)**, partially filled seed **(B)**, flatten shrunken seed **(C)**, partially filled yellow seed **(D)**, sprouting fully filled seed **(E)**, sprouting flatten shrunken seed **(F)**. Scale bars represent 1 mm. **(G)** Graph displaying the number of viable seeds per silique in DH12075, Topas, and Westar cultivars at CT (green), MT (yellow), and HT (red). **(H)** Graph displaying 100-seed weight in DH12075, Topas, and Westar cultivars at CT (21/18°C, green) and MT (28/18°C, yellow). The data **(G,H)** are presented in boxplots. The box represents the interquartile range, and the black line inside the box represents the median. Dots represent outliers. Boxes with the same letters (a, b, and c) within each cultivar do not differ significantly (*p* < 0.05).

**Figure 6:**
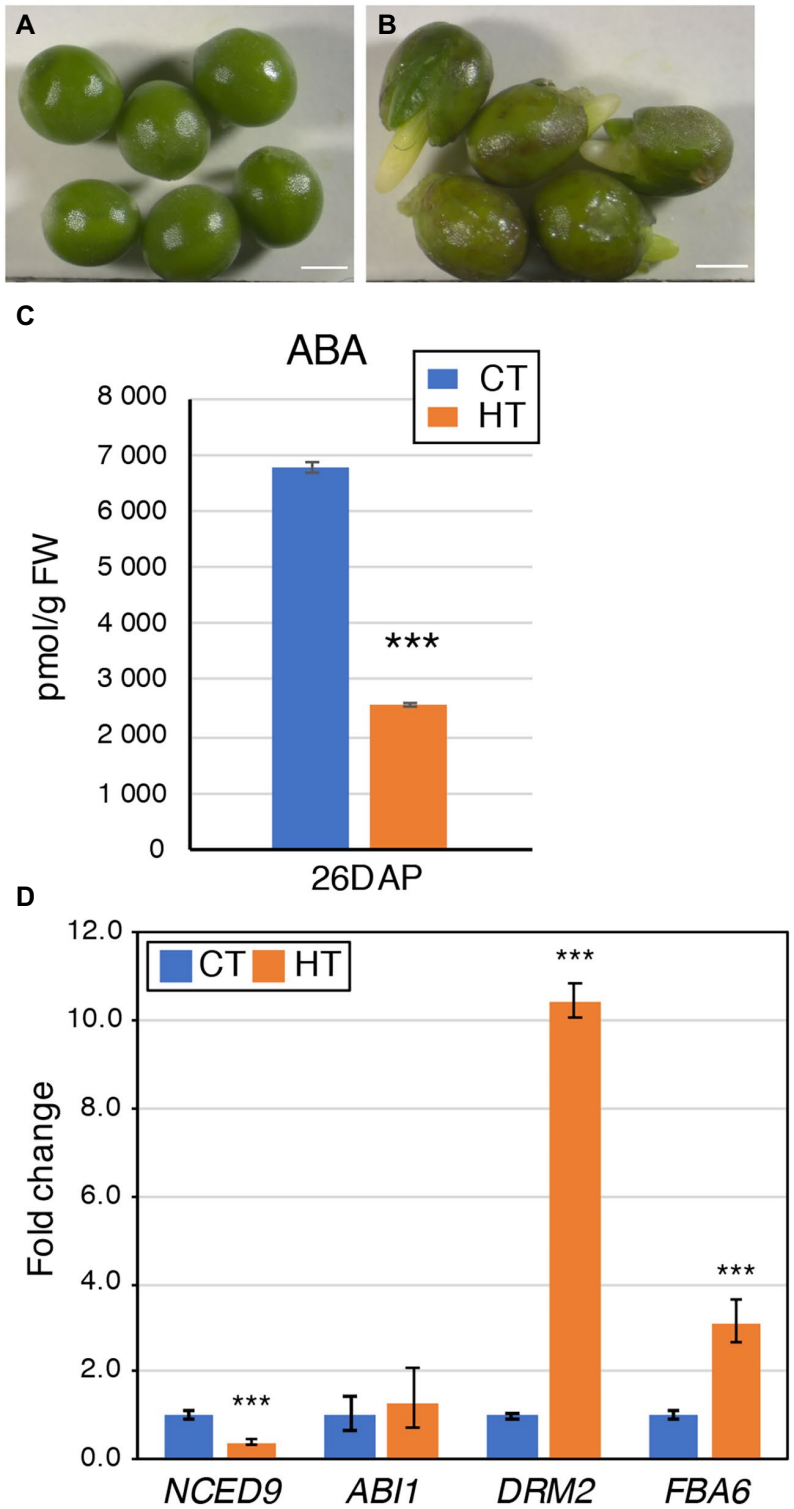
Pre-harvest sprouting seed phenotypes may be linked to a decrease in ABA levels. **(A,B)** Topas 26 DAP seeds grown at CT **(A)** and HT **(B)**. Notice the sprouting of the seeds **(B)**, with the emergence of either root or cotyledons. Scale bars represent 1 mm. **(C)** Graph showing the ABA levels (in pmol/g FW) in Westar 26 DAP seeds grown at CT and HT. Shown is the average ± SD. Asterisks indicate a statistically significant difference in HT in a paired Student’s *t*-test (*t*-test; *** corresponds to value of *p* < 0.001). Source data are presented in Supplementary Table S4. **(D)** Expression analysis by RT-qPCR of *BnaNCED9*, *BnaABA1*, *BnaFBA6*, and *BnaDRM2* in Westar 26 DAP seeds. The graph displays the fold changes in expression between CT (blue) and HT (orange). Asterisks indicate a statistically significant difference in HT in a paired Student’s *t*-test (*t*-test; *** corresponds to value of *p* < 0.001). Primers and LOC information are presented in Supplementary Table S1.

The number of viable seeds per silique and seed phenotypes were examined after harvest (Figure 5). Three categories of seeds: fully filled (Figure 5A), partially filled/shrunken (Figures 5B–D), and germinated/sprouted (Figures 5E,F), were considered viable seeds. Both regimes with elevated temperatures severely affected the viable seed yield, with a significant decrease at MT, and a critical one at HT, resulting in almost no viable harvested seeds (Figure 5G). The Topas cultivar has the highest number of germinated seeds before harvest (PHS). It was evident that part of those seeds germinated even before maturation (Figures 5E,F, 6A,B; Table 3).

**Table 3:** Comparison of pre-harvest germination of mature and dry seeds (percentage of total seeds for one plant).

	21°C		34°C	
	26 DAP	After harvest	26 DAP^*^	After harvest
DH12075	0	0.53	2.8–13.2	11.9
Topas	0	0.7	30.4–73.4	40.8
Westar	0.45	0.22	3.8–7.9	4.1

* 95% CI.

Fully and partially filled seeds were investigated for seed weight characteristics. In DH12075 and Westar, the 100-seed weight decreased significantly in MT conditions (Figure 5H). HT was not chosen for this experiment due to a poor yield of fully developed seeds under these temperature conditions. A decrease in weight was not identified in Topas. With the highest number of seeds per silique and stable seed weight, Topas could be considered the best yield provider under MT and HT growth conditions.

### Cultivar Specific Pre-harvest Sprouting Occurred at HT and Coincided With a Decreased ABA Content

Based on the mature seed phenotyping analysis, many Topas seeds displayed a sprouting phenotype, which appeared before seed maturation (Figures 6A,B; Table 3). In these seeds, either the embryo emerged from the seed coat, or the seed coat ruptured due to the embryo pressure from 20 to 35 DAP. PHS rarely occurred in CT (less than 1% for all cultivars), but its rate is significantly higher in HT (30.4%–73.4% in Topas). To a lesser extent, it was also observed in Westar (3.8%–7.9%) and DH12075 (2.8%–13.2%) in HT (Table 3). The occurrence of PHS corresponds with the percentage of germinated dry seeds after harvest. Sprouting seeds are not viable because they did not progress into the seed maturation phase.

**Figure 7:**
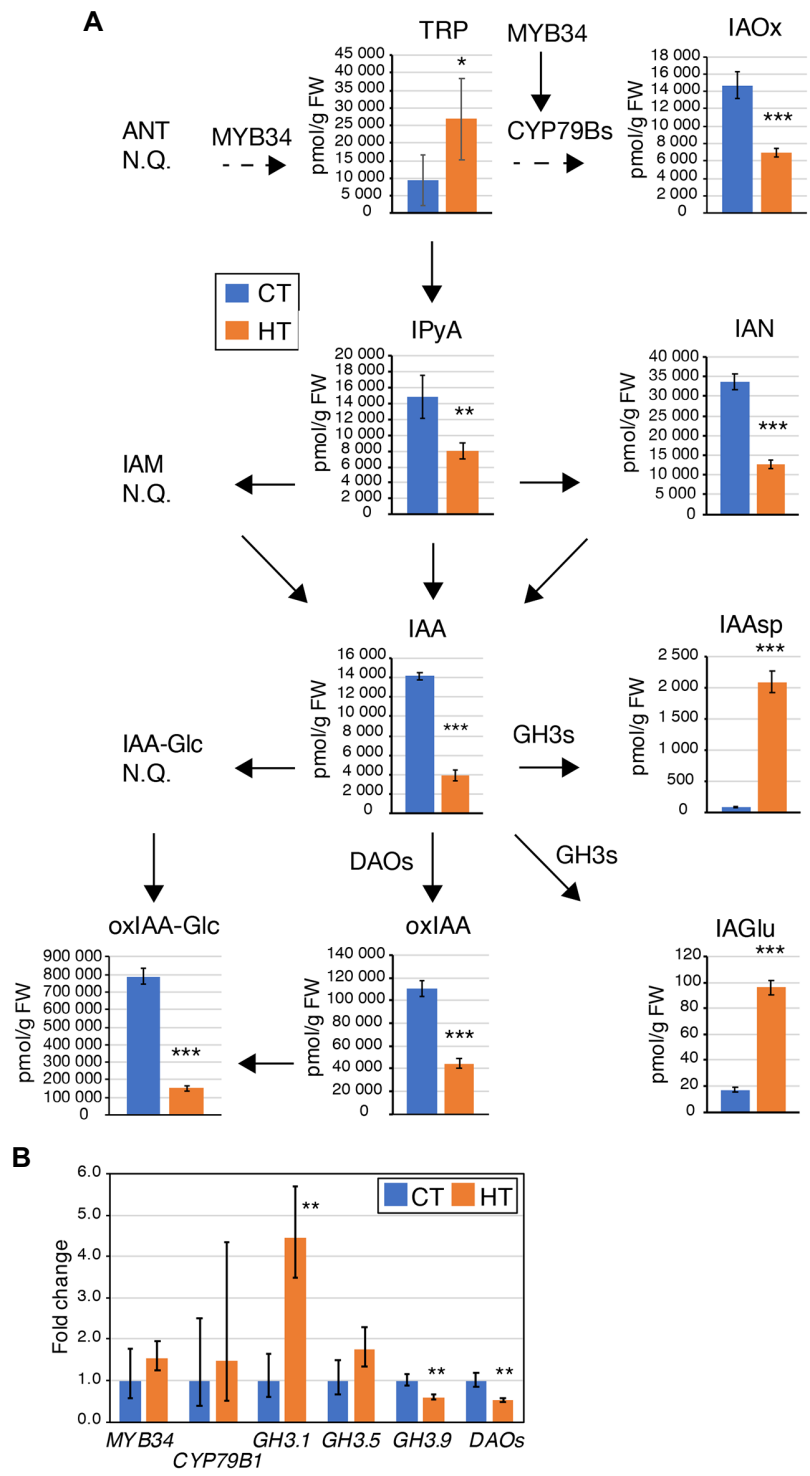
Differences in auxin and auxin metabolites levels and enzyme expression induced at HT in Westar 26 DAP seeds. **(A)** Graphs displaying the levels (pmol/g FW) of tryptophan (TRP), indole-3-acetaldoxime (IAOx), indole-3-pyruvic acid (IPyA), indole-3-acetonitrile (IAN), indole-3-acetic acid (IAA), IAA-aspartate (IAAsp), IAA-glutamate (IAGlu), 2-oxoindole-3-acetic acid (oxIAA) and oxIAA-glucose (oxIAA-Glc). Anthranilate (ANT), indole-3-acetamide (IAM), IAA-glucose (IAA-Glc) were not quantified (NQ). Measurements were done in two biological replicates, five technical replicates in Westar 26 DAP seeds from plants grown at CT (blue) and HT (orange). Shown is the average ± SD of one of the two biological replicates. Asterisks indicate statistically significant difference in HT in a paired Student’s *t*-test (*t*-test; *, **, and *** correspond to value of *p* 0.05 > *p* > 0.01, 0.01 > *p* > 0.001, and *p* < 0.001, respectively). Arrows indicate the direction of the biosynthesis pathway (plain arrow, direct reaction; dashed arrow, multiple-step enzymatic reactions). The names closed to the arrows indicate the enzymes involved in the reactions and whose gene expression was tested **(B)**. Source data are shown in Supplementary Table S3. **(B)** Expression analysis by RT-qPCR of *BnaMYB4*, *BnaCYP79Bs*, *BnaGH3.1*, *BnaGH3.5*, *BnaGH3.9*, and *BnaDAOs* in Westar 26 DAP seeds. The graph displays the fold changes in expression between CT (blue) and HT (orange). Asterisks indicate statistically significant difference in HT in a paired Student’s *t*-test (*t*-test; ** corresponds to value of *p* 0.01 > *p* > 0.001). Primers and LOC information are presented in Supplementary Table S1.

Growth at HT altered hormonal levels and metabolites content in 26 DAP seeds. Higher TRP content with lower levels of direct auxin precursors (indole-3-pyruvic acid (IPyA), indole-3-acetaldoxime (IAOx), and IAN) was observed in HT seeds (Figure 7A). IAA levels are also decreased in 26 DAP seeds. Furthermore, a shift from the oxidative auxin degradation by the DAO enzymes toward the production of amino acid-auxin conjugates was observed (less 2-oxoindole-3-acetic acid, oxIAA, and oxIAA-Glc, more indole-3-acetic acid-aspartate, IAAsp, and indole-3-acetic acid-glutamate, IAGlu). It was confirmed by an expression analysis showing downregulation of *DAO*s genes and upregulation of some *GH3* genes (Figure 7B). Moreover, 26 DAP seeds are in the seed maturation stage, preparing for dormancy. ABA is known to promote dormancy in many crops (Groot and Karssen, 1992; Lefebvre et al., 2006; Huang et al., 2016). The significant decrease in ABA levels in 26 DAP seeds from HT (Figure 6C; Supplementary Table S4) indicates that HT limits seed dormancy progression by reducing the *NCED9* expression (Figure 6D). Lower ABA levels may explain the PHS phenotype when the embryo continues to grow, breaks the seed coat, and finally desiccates during fruit ripening. Accordingly, *BnaFBA6* and *BnaDRM2*, genes involved in sugar metabolism and dormancy release, were significantly upregulated at HT in 26 DAP seeds (Figure 6D). In addition, the genes involved in alternative splicing, *SLU7* and *SCL30A*, were significantly upregulated, while *RNPII30C* is downregulated in 26 DAP seeds (Figure 4I).

### Seed Development at MT Results in Altered Seedling Morphology

**Figure 8:**
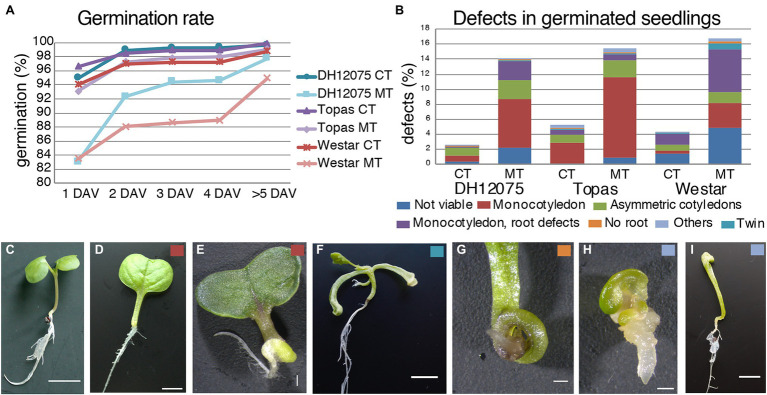
Seed development at high temperatures affects seedling viability. **(A)** Graph displaying the cumulative percentage of seed germination at 1, 2, 3, and 4 day after vernalization (DAV), and later for seeds produced by DH12075, Topas, Westar plants grown at CT and MT. **(B)** Percentage of defective seedlings per categories of defects: not viable (dark blue), with one cotyledon (red), asymmetrically positioned cotyledons (green), twin seedlings (turquoise), with one cotyledon, and defective root (purple), without any root (orange) and other categories (light blue). The analysis was performed on seedlings from seeds produced by DH12075, Topas, Westar plants grown at CT and MT. **(C–I)** Range of observed seedling phenotypes. A wild-type seedling is shown **(C)**. Seedlings **(D,E)** are with one cotyledon (red). **(F)** The seedling is with two roots and two shoots (turquoise). **(G)** Zoom in of a seedling without root (orange). **(H,I)** Are shown seedlings from the other categories (light blue). The small colored square in the upper right corner refers to the categories. Scale bars represent 10 mm.

Fully and shrunken-filled seeds were investigated for germination. Seedling viability and appearance were monitored for seeds collected in CT and MT. In general, most of the seeds (>80%) germinated 1 day after vernalization, regardless of the temperature, the seeds developed, reaching >94% of germination rate after the 4th day. Within this range, a reduction in germination was notable for DH12075 (2%) and Westar (4%) seeds (Figure 8A).

In CT, the rate of defective seedlings was 2.1% for DH12075, 5.3% for Topas, and 4.3% for Westar (Figure 8B). In MT, the percentage of defective seedlings increased to 12% for DH12075, 15.4% for Topas, and 16.8% for Westar (Figure 8B). Several types of defects were detected, from which some may partially be the consequence of the defects observed during the early embryo phenotyping analysis (Figures 4, 8C**–** I). We observed viable seedlings with unequal shape of cotyledons (0.67%–1.1% in CT, 1.5%–2.5% in MT) or only one cotyledon (0.44%–2.7% in CT, 3.2%–10.7% in MT, Figures 8D,E). This defect was in some cases combined with either the absence of a root or shoot apical meristem or both (Figures 8G,I). Those seedlings completely stopped growing or just delayed their development until a new root or shoot was developed, thanks to the high plasticity of plant regeneration. Other detected phenotypes were, for example, seedlings with three cotyledons and secondary seedlings occurring in less than 1% of germinated seeds (Figure 8F).

### Seed Quality Is Mildly Affected by MT in All Three Cultivars

**Figure 9:**
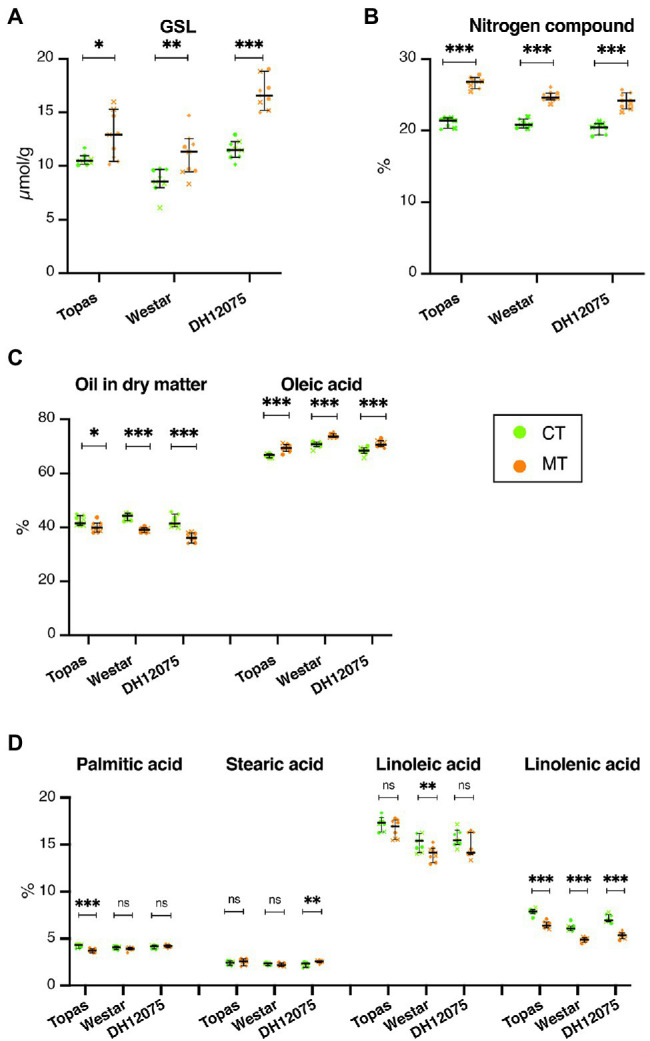
Differences in Glucosinolates (GSL), Nitrogen, and seed oil levels induced at MT. **(A–D)** Graphs are displaying the levels of glucosinolates (GSL in 9% humidity, μmol/g; **A**), nitrogen compound (%; **B**), total oil in dry matter (%) and oleic acid (% of total oil; **C**), palmitic acid (% of total oil), stearic acid (% of total oil), linoleic acid (% of total oil) and linolenic acid (% of total oil; **D**) at CT (green) and MT (orange) in Topas, Westar and DH12075 cultivars. The data from three biological replicates are plotted (•, replicate 1; ×, replicate 2; ♦, replicate 3). The horizontal line represents the median with 95% CI. Asterisks indicate statistically significant difference in MT in a paired Student’s *t*-test (*t*-test; *, **, and *** correspond to value of *p* 0.05 > *p* > 0.01, 0.01 > *p* > 0.001, and *p* < 0.001, respectively). Source data are found in Supplementary Table S6. The measurements were done in three biological replicates, each in three technical replicates.

As *Brassica napus* is an oilseed crop, the quality and composition of oil and the content of glucosinolates and nitrogen compounds were tested in seeds. In general, the oil content was reduced by growth at MT temperature regime by 2%–6% in all the cultivars in MT compared to CT (Figure 9; Supplementary Table S6). The highest decrease was found in DH12075 cultivar from 42.27% (CT) to 36.24% (MT) in dry matter (Figure 9C; Supplementary Table S6). Changes were also detected in oil quality and quantity of unsaturated fatty acids (Figures 9C,D; Supplementary Table S6). The main compound in rapeseed oil is oleic acid (C18:1), which was increased by ~3% in MT at the expense of desaturated linoleic acid (C18:2) and linolenic acid (C18:3), which decreased in average by 0.86% and 1.49%, respectively. These changes correlate with the changes in the desaturation activity of oleic acid measured by ODP: decreased in average by 2.71% in all cultivars at MT (Supplementary Table S6). Moreover, the desaturation activity of linoleic acid measured by LDP decreased in average by 3.75% with temperature in all cultivars, indicative of the accumulation of linoleic acid over linolenic acid at MT (Supplementary Table S6). Contents of palmitic and stearic, in rapeseed oil are low (<5%), and their changes are not biologically relevant (Figure 9D).

Seeds also contain more proteins measured as levels of nitrogen compounds. Protein levels are increased by more than 4.42% in MT (Figure 9B; Supplementary Table S6). Growth at MT increased the glucosinolate levels by 2.17–5.29 μmol/g in MT. In total numbers, Topas exhibits the lowest increase in glucosinolates (+2.17 μmol/g with 12.77 μmol/g in MT). On the other hand, in DH12075, the level of glucosinolates increased to 16.80 μmol/g (Figure 9A). However, the amount of GSL did not reach harmful concentrations for consumption.

## Discussion

In this study, phenotypic responses to elevated temperatures were described on the whole flowering plant level. Plants reacted to HT during reproductive development by prolonging the growth of the main stem and their flowering time and developing more branches to compensate for the lower pollination and fertilization rates and increased seed abortion. Thus, based on the prolonged flowering duration, we hypothesized an alteration in the photoperiod or maturation-related regulation of hormonal levels during plant development. We observed that *PHYA*, involved in photomorphogenesis (Seaton et al., 2018), is downregulated at HT. Similarly, *ELF4*, stabilizing ELF3 to regulate flowering negatively (Doyle et al., 2002; Lin et al., 2019), is strongly repressed in pistils at HT. The Evening Complex, comprising ELF3 and ELF4, binds less strongly the promoter of the thermomorphogenesis-promoting transcription factor PIF4 when temperature increases, derepressing its transcription (Ezer et al., 2017). The higher expression in pistils of *FLOWERING LOCUS T*, a target of PIF4 at warm ambient temperature (Kumar et al., 2012), is consistent with the downregulation of *ELF4*. This expression analysis suggests that high temperatures may activate thermomorphogenesis by reducing the amount of the Evening Complex in leaves and pistils. As previous studies applied heat stress for limited periods, it hardly showed the changes on the whole plant level, although partial hints in accordance with our results were published. Indeed, HT increased plant growth and their above-ground biomass regardless of the genotype (Chen et al., 2020a). And significantly higher production of lateral inflorescences led to higher production after stress release and returned to control conditions (Young et al., 2004). Both studies showed that these changes are not specific to the cultivars used in our study but could be broadly applied. Comparing the effects of elevated temperatures in the three studied cultivars, Topas appears to be more resistant and DH12075 more sensitive to the temperature effects than Westar when looking at flower production and flowering time (Figure 1; Supplementary Figure S2).

We did not find any biologically significant changes in pollen viability, which could cause a decrease in fertilization rate and seed number per pod. These findings do not correspond with published data of reduced pollen viability after 7-day heat treatment in *B. napus* and *B. rapa* (Young et al., 2004; Annisa et al., 2013; Chen et al., 2021). Our cultivation setup includes lower night temperatures (18°C in all conditions) and ramping up and down to stress temperatures during the day. The pollen development may benefit from the colder night temperatures to cope with the stress conditions. However, despite pollen grain viability, all three cultivars had a significantly reduced seed set in a dose-dependent reaction to warm temperature. This significant decrease in fertilization rate (occurrence of unfertilized ovules) may be caused by the infertility of the ovules, lower pollen germination (Young et al., 2004), lower growth of pollen tubes through the transmitting tract, and defects in pollen-ovule communication leading to low attractivity of the pollen tube towards the ovule. In addition to a low fertilization rate with increasing temperatures, some of the developed seeds were aborted in a dose-dependent manner. Only the DH12075 cultivar was resistant to warm temperatures on early seed abortion. Aborted seeds due to elevated temperature were previously noticed without further investigations (Chen et al., 2020a). Early seed abortion is most likely a consequence of the severe patterning defects of the embryos. DH12075 and Westar produced less viable seeds per silique and more seeds with defective embryo development in HT. In comparison, siliques of Topas produced more seeds, and embryo development was less affected at HT. However, HT strongly affected Topas seed maturation, with 30%–73% seeds affected by PHS. As a result of the HT-dependent reduced fertilization rate, early seed abortion, PHS, asynchronous seed development, and other issues with seed maturation, the number of viable seeds per silique was severely decreased: less than 5 seeds per silique for DH12075 and Westar, and less than 10 seeds per silique for Topas. The reduction of seed number was combined with a mild reduction in the seed weight in DH12075 and Topas only. A comparable effect was observed in rice (Wu et al., 2016). Angadi et al. (2000) suggested that the optimal daytime temperature for *B. napus* is closer to 28°C instead of 20°C or 35°C. Overall, the high-temperature effect on seed development and production is multifactorial.

In our knowledge, the dynamics of zygotic embryo development in higher temperatures have not yet been studied in flowering plants. HT accelerates early embryo development, which may cause the misregulation of essential signaling pathways. The described embryonic defects indicate irregularities in cell division patterns in HT compared to CT. Supernumerary suspensor cells were observed at HT in elongated globular embryos. Defective embryos present imprecise determination of cell fate in suspensor cells with what may be the formation of secondary embryonic mass in the suspensor. Proembryos were misshaped with ill-defined root apical pole and misregulated apical organ formation. This defective development affects 15%–40% of embryos in HT. Supernumerary suspensor cells and loss of polarity in embryonic mass were also observed after thermal stress during somatic embryogenesis in radiata pine (Castander-Olarieta et al., 2019). Auxin is essential for regulating embryonic morphogenesis with the establishment of an apical-basal polarity (Benková et al., 2003; Friml et al., 2003), maintaining of suspensor cell fate (Rademacher et al., 2012; Radoeva et al., 2019), endosperm development (Figueiredo et al., 2015), and seed coat development (Figueiredo et al., 2016). Decreased auxin levels in HT may alter the synchronous development of the embryo, endosperm, and seed coat, therefore explaining the higher abortion rate. Also, the observed embryonic defects (altered morphology) may be explained by lower auxin levels, transport and signaling. Misspecification of the root apical meristem was identified in the *taa1 tar1 tar2* and *yuc1 yuc4* auxin biosynthetic mutants, *aux/lax* auxin transport mutants, and the *monopteros* auxin signaling mutant (Hardtke and Berleth, 1998; Robert et al., 2013, 2015). Extra horizontal and vertical divisions of suspensor and missing cotyledons were described in the *tir1 afb2 afb3 afb5* quadruple mutant (Prigge et al., 2020). Suspensor cell identity was disturbed in *iaa10* mutant leading to missing root tissue (Rademacher et al., 2012) or forming secondary embryos (Radoeva et al., 2019). Maintenance of both embryonic and suspensor cell fate is essential for correct development. In CT, 5-DAP-old seeds contain embryos from 2-cell to 8-cell stages, which requires the transport of auxin from the seed integuments for its proper development (Robert et al., 2018). In HT, 5-DAP-old seeds bear globular embryos, which produce the auxin needed for its morphogenetic development (Robert et al., 2013). We hypothesized that HT alters auxin distribution and/or signaling in suspensor and embryo proper, resulting in the described embryonic defects. Indeed, auxin profiling in young seeds (5 DAP, globular stage) indicated that auxin production is reduced with a lower amount of TRP, the precursor of IAA, and lower levels of IAA. Some of the patterning defects occurring during embryo development were visible in germinated seedlings, so those embryos were viable and went through the seed maturation phase. Other defects increased the seed abortion penetrance during later stages of seed development. We did not observe a high reduction in the germination rate of fully and partially filled seeds suggesting that imperfectly developed seeds and severely defective embryos underwent abortion during development. Still, we found up to 16.8% defective seedlings at MT (against 5.3% at CT). Most of these seedlings were viable, and despite slower growth, they progressed into normal development. The defects observed in seedlings are connected to the observed embryonic defects and, most probably, the identified changes in hormonal levels.

An additional factor to proper embryo patterning is the pre-mRNA splicing required for the function of many essential genes. We observed the change in expression of three genes involved in the spliceosome complex *SLU7*, *SCL30A*, and *HSP70* (annotated in Genbank as the subunit 37c of the RNA polymerase II, *RNPII37C*), indicating that HT misregulated the spliceosome activity in seeds (and other tissues). Generic or specific transcript aberrant splicing activity may also explain the decreased fertilization rate and increased embryo defects and seed development defects at HT. Indeed, mutations in the spliceosome complex protein *PRP8A* and *CWC15*, for example, led to embryonic defects very similar to the ones we observed (Kulichová et al., 2020; Slane et al., 2020).

In our study, the reduced quality of the harvest was partially caused by PHS initiated by HT. It was noticed in all three studied cultivars, and most severely in Topas (30.4%–73.4%), where the embryo continues to grow, breaks the seed coat, and finally desiccates during fruit ripening. Seed sprouting and lower ABA content in HT indicate that seeds did not progress into the dormancy phase but continued directly to germination. Primary dormancy in *B. napus* coincides with a peak of ABA between 30 and 40 DAP, decreasing rapidly to 0%–15% in the next 2–3 weeks (Huang et al., 2016). Therefore, our experiment showed that the embryos accelerated development caused seed sprouting before the expected ABA accumulation. The dormancy induction in developing seeds is achieved by embryonic ABA production (Groot and Karssen, 1992). The production in maternal tissue can only partially induce dormancy in Arabidopsis in the absence of zygotic ABA (Kanno et al., 2010). *NCED6* and *NCED9* are the main ABA biosynthetic genes expressed preferentially in developing seeds (Lefebvre et al., 2006). Disruption of their activity reduced seed dormancy in *Arabidopsis*. Our expression analysis identified a correlation between the reduced *NCED9* expression levels and the reduced ABA content in 26 DAP seeds at HT. Furthermore, HT treatment caused fine-tuning changes in auxin degradation by reducing auxin levels in 26 DAP seeds. Levels of oxIAA are decreased by half (even more for oxIAA-Glc), which are compensated by an increase in conjugative degradation by GH3 proteins, a phenomenon which has also been described in the Arabidopsis *dao1-1* mutant line (Mellor et al., 2016). Auxin has been shown to act upstream of ABA in controlling seed dormancy (Liu et al., 2013). Auxin signaling controls the expression of ABA signaling genes involved in the progression and maintenance of seed dormancy. Recently, it has been shown that an accumulation of IAM in plant tissue impairs growth and seed development and represses temperature stress-related genes (*HSFA2*, *HSFA3*; Sánchez-Parra et al., 2021). Moreover, a crosstalk between the levels of IAM (and AMIDASE1 activity) and ABA has been identified (Pérez-Alonso et al., 2021). Therefore, in 26 DAP seeds at HT, seed sprouting may be induced by reduced ABA levels and a reduced auxin input on ABA signaling. The absence of dormancy is characterized by the mobilization of seed reserves for seed germination. Studies showed that *FBA6*, involved in the glycolysis pathway, is upregulated in seedlings in response to sugar treatments and heat stress in Arabidopsis (Lu et al., 2012), which might correlate with the expected increased sugar metabolism and partial loss of dormancy in HT 26 DAP seeds showing PHS phenotype (for which DRM2 may be the marker).

Surprisingly, a decrease in IAN levels was detected in Westar seeds. A general tendency of higher GSL content in seeds in response to higher temperatures was observed in our study for Topas, Westar and DH12075 in MT, contrary to previous studies (Aksouh-Harradj et al., 2006; Rao et al., 2021). On the other hand, such an increase was not significant in drought stress (Hatzig et al., 2018). Moreover, mutants in GSL metabolism exhibited lower levels of the heat-shock stress protein 90 (HSP90) and reduced tolerance to elevated temperatures (Ludwig-Müller et al., 2000). Noteworthy, the cultivars used in this study are low-glucosinolate varieties. Therefore, the observed GSL changes may be small or different compared to the high-temperature response in varieties with high-GSL varieties.

In general, plants mostly react to stress by increasing their ABA production to regulate their water balance, reduce desiccation, and, in the longer term, regulate the senescence of different plant organs. Surprisingly, we found that ABA levels were decreased in non-pollinated pistils and 26 DAP seeds, while they remained unchanged in 5 DAP seeds in HT compared to CT. It may suggest that Brassica plants have minor abilities to protect themselves in long-term heat stress and, therefore, led to a high abortion rate, decreased yield, and reduced dormancy in the case of 26 DAP seeds in Topas. These results contrast with research performed on pea seeds (Kaur et al., 2020) and rice panicles (Wu et al., 2016).

Overall, Topas cultivar is not much affected by growth at HT. Topas presented a reduced fertilization rate at 54%, the highest among the three cultivars, but was highly affected by seed abortion and PHS (Table 4). DH12075 was the most affected in overall plant growth (flowering time, number of produced branches, and flowers). However, DH12075 had the lowest seed abortion rate, with a fertilization rate of 48%. Nevertheless, DH12075 had a low viable seed production with the highest rate of defective embryos (Table 4). Westar manifested an intermediate reaction to flowering growth at warm ambient temperature among the three cultivars (Table 4). Westar had a reduced number of ovules per pistil, the lowest fertilization rate (45%), an intermediate seed abortion rate, and a defective embryo rate, resulting in a low viable seed set. Because all three cultivars were low GSL producers, warm ambient temperature affected GSL to a low extent by up to a 5% increase, below the rate for unhealthy consumption (20 μmol/g). And oil production was only reduced by up to 6% (Table 4).

**Table 4:** Comparison of the impact of warm ambient temperatures in the three studied cultivars.

Phenotypes	DH12075	Topas	Westar
Number of produced flowers (HT)	Sensitive (higher)	Resistant (~unaffected)	Intermediate
Flowering time (HT)	Sensitive (longer)	Resistant (slightly reduced)	Intermediate (slightly longer)
Apical dominance (HT)	Reduced	Unaffected	Reduced
Pollen viability	~95% Viable	~95% Viable	~95% Viable
Number of ovules per pistil (HT)	Unaffected	Unaffected	Mild reduction
Fertilization rate (HT)	48%	54%	45%
Seed abortion (HT)	<1%	15%	12%
Defective embryos (HT)	32%	19%	25%
PHS	Intermediate (3%–13%)	Sensitive (30%–73%)	Resistant (4%–8%)
Viable seed set (HT)	~1 seed per silique	~6 seeds per silique	~2 seeds per silique
Seed quality (MT)—GSL	+5%	+2%	+2%
Seed quality (MT)—Oil	−6%	−2%	−5%
Seed weight (MT)	Mild reduction	Mild reduction	No difference
Seed germination	Delayed	Unaffected	Delayed

In summary, elevated temperature influences the reproductive growth of *B. napus* in different manners. Our growth setup allowed for the monitoring of thermomorphogenesis in flowering and seed-producing rapeseeds, contrary to previous studies focused on short heat shocks or short temperature treatments. Seed development is negatively affected, leading to a higher abortion rate, probably due to misregulated embryo development and auxin signaling. We report the impact of high temperatures on embryonic patterning: supernumerary suspensor cells, secondary embryo development, and apical-basal polarity defects. HT modified auxin and ABA homeostasis during morphogenic and maturation embryonic phases. Decreased ABA levels during maturation are in connection to a higher PHS rate. Our observations pinpointed several aspects that will require more in-depth analysis to understand seed development in response to suboptimal growth temperatures. We believe our work will be beneficial for better understanding of how plants respond to long-term warm ambient temperature, which paves the way to generating thermo-resilient crops, thus improving crop yield.

## Data Availability Statement

The raw data supporting the conclusions of this article will be made available by the authors, without undue reservation.

## Author Contributions

KM: investigation (all), formal analysis, visualization, writing-original draft preparation, and writing-review and editing. UP and MŠ: investigation (phenotyping, RT-qPCR) and writing-review and editing. IS: formal analysis (statistical analysis) and visualization. AP and ON: investigation (hormone profiling). LE: investigation (GSL, nitrogen, and oil measurements). HR: conceptualization, funding acquisition, project administration, supervision, visualization, writing-original draft preparation, and writing-review and editing. All authors contributed to the article and approved the submitted version.

## Funding

This work was supported by the Czech Science Foundation (project no. 19-05200S) to HR, from the Ministry of Education, Youth and Sports of the Czech Republic with the European Regional Development Fund-Project “SINGING PLANT” (no. CZ.02.1.01/0.0/0.0/16_026/0008446).

## Conflict of Interest

The authors declare that the research was conducted in the absence of any commercial or financial relationships that could be construed as a potential conflict of interest.

## Publisher’s Note

All claims expressed in this article are solely those of the authors and do not necessarily represent those of their affiliated organizations, or those of the publisher, the editors and the reviewers. Any product that may be evaluated in this article, or claim that may be made by its manufacturer, is not guaranteed or endorsed by the publisher.

## Figures and Tables

**Table:** 

ANT	Anthranilate
ABA	Abscisic acid
ANOVA	Analysis of variance
CT	Control-temperature growth regime
DAP	Days after pollination
DAV	Days after vernalization
EG	Early globular
FT	Flowering time
FT-NIR	Fourier transform near-infrared
GC/FID	Gas chromatograph-flame ionization detection
GSL	Glucosinolates
HPLC	High performance liquid chromatography
HS	Heat stress
HSF	Heat stress transcription factor
HSP	Heat shock protein
HT	High-temperature growth regime
IAA	Indole-3-acetic acid
IAAsp	Indole-3-acetic acid-aspartate
IAA-Glc	Indole-3-acetyl-1-O-ß-d-glucose
IAGlu	Indole-3-acetic acid-glutamate
IAM	Indole-3-acetamide
IAN	Indole-3-acetonitrile
IAOx	Indole-3-acetaldoxime
IPyA	Indole-3-pyruvic acid
LDP	Linoleic desaturation proportion
LG	Late globular
LMS	Length of main flowering stem
LOC	Gene locus name
LOD	Limit of detection
MG	Mid-globular
MIQE	Minimum information for publication of quantitative real-time PCR Experiments
MT	Mid-temperature growth regime
NB	Number of primary branches
NF	Number of flowers on the main stem
NIR	Near-infrared
NL	Number of leaves
NO	Number of ovules
NQ	Not quantified
NS	Number of seeds
ODP	Oleic desaturation proportion
oxIAA	2-Oxoindole-3-acetic acid
oxIAA-Glc	2-Oxoindole-3-acetyl-1-O-ß-d-glucose
PHS	Pre-harvest sprouting
RGR	Relative growth rate
RSD	Relative standard deviation
RT-qPCR	Reverse transcription-quantitative polymerase chain reaction
SD	Standard deviation
SL	Silique length
TRP	Tryptophan
μSPE	Micro solid-phase extraction
UV–VIS	Ultraviolet and visible light wavelengths
