# Supplementary material for: Long-Term High-Temperature Stress Impacts on Embryo and Seed Development in Brassica napus
Source: Front Plant Sci. 2022 Apr 22;13:844292. doi: 10.3389/fpls.2022.844292 (PMC9075611; doi:10.3389/fpls.2022.844292)
Supplement: Supplementary file 1 [file Data_Sheet_1.PDF]

Supplementary Material

1 Supplementary Figures and Tables

Figure S1. Setup of growth temperatures in greenhouse chambers and experimental design.
Figure S2. High temperatures affect growth parameters of Brassica flowering plants.
Figure S3. The number of ovules and the pollen development are not affected by high temperatures.
Figure S4. Embryo development is accelerated by high temperatures.
Figure S5. High temperatures affect embryo development. Original pictures presented in Figure 4.
Figure S6. Silique growth rate is reduced at elevated temperatures.
Table S1. Primers used in qPCR analysis and LOC number of amplified genes.
Table S2. Pearson correlation coefficient between the length of the main inflorescence stem, the flowering time, and the number of flowers.
Table S3. Auxin and auxin metabolites measurements (source data of Figures 2 and 7).
Table S4. ABA measurements (source data of Figure 3).
Table S5. Pearson correlation coefficient between the siliques growth rate, the seed number per silique, and the growth temperatures.
Table S6. Glucosinolates, nitrogen, and seed oil measurement (source data of Figure 9).

1.1 Supplementary Figures

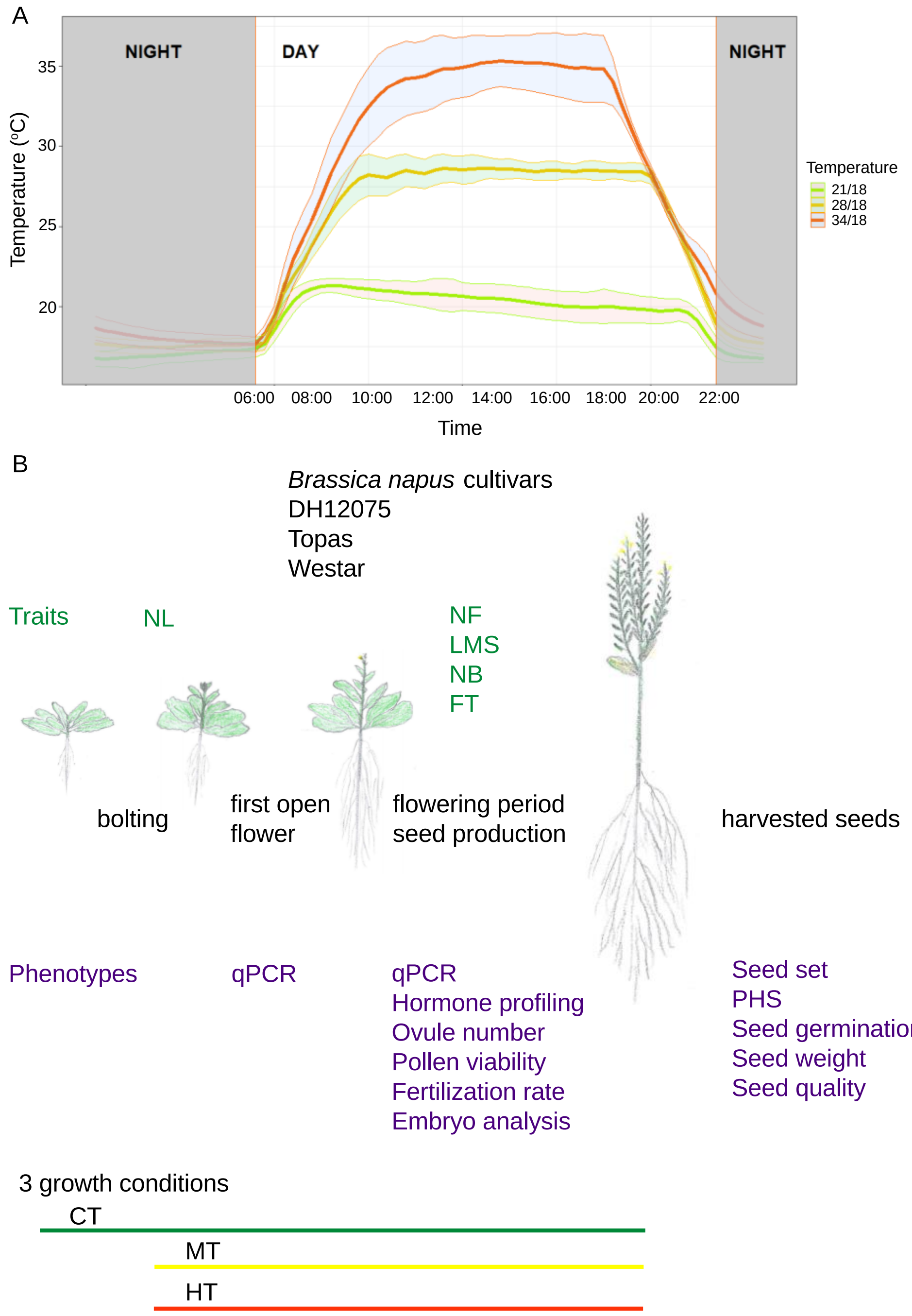

Supplementary Figure 1. Setup of growth temperatures in greenhouse chambers and experimental design. (**A**) Three conditions were selected for the temperature stresses and control conditions. Temperature condition for control (CT, 21/18, green), mid (MT, 28/18, yellow), and high temperature (HT, 34/18, red) chambers were set to 21 °C, 28 °C, and 34 °C, respectively, with ramping of the temperature up and down by 4 °C per hour from night temperatures set at 18 °C for all conditions. Day period was set between 6:00 and 22:00 (light grey). The graph shows the mean temperatures (bold line)

± 95 % confidence interval in each greenhouse chamber measured throughout the experiment. (**B**) Experimental design. Plants from three *Brassica napus* cultivars (DH12075, Topas, and Westar) were grown until bolting at CT growth temperatures, then kept at CT or moved to MT or HT until seed harvest. Traits are measured at bolting (NL, number of leaves) and during the flowering period and seed production: NF (number of flowers), LMS (length of the main flowering stem), NB (number of branches,) and FT (flowering time). Phenotyping experiments are also performed: expression analysis by qPCR on leaves, pistils, and seeds, hormonal profiling, ovule number, pollen viability, fertilization rate, embryo phenotyping, seed set, PHS (pre-harvest sprouting), seed germination assay, seed weight, and quality.

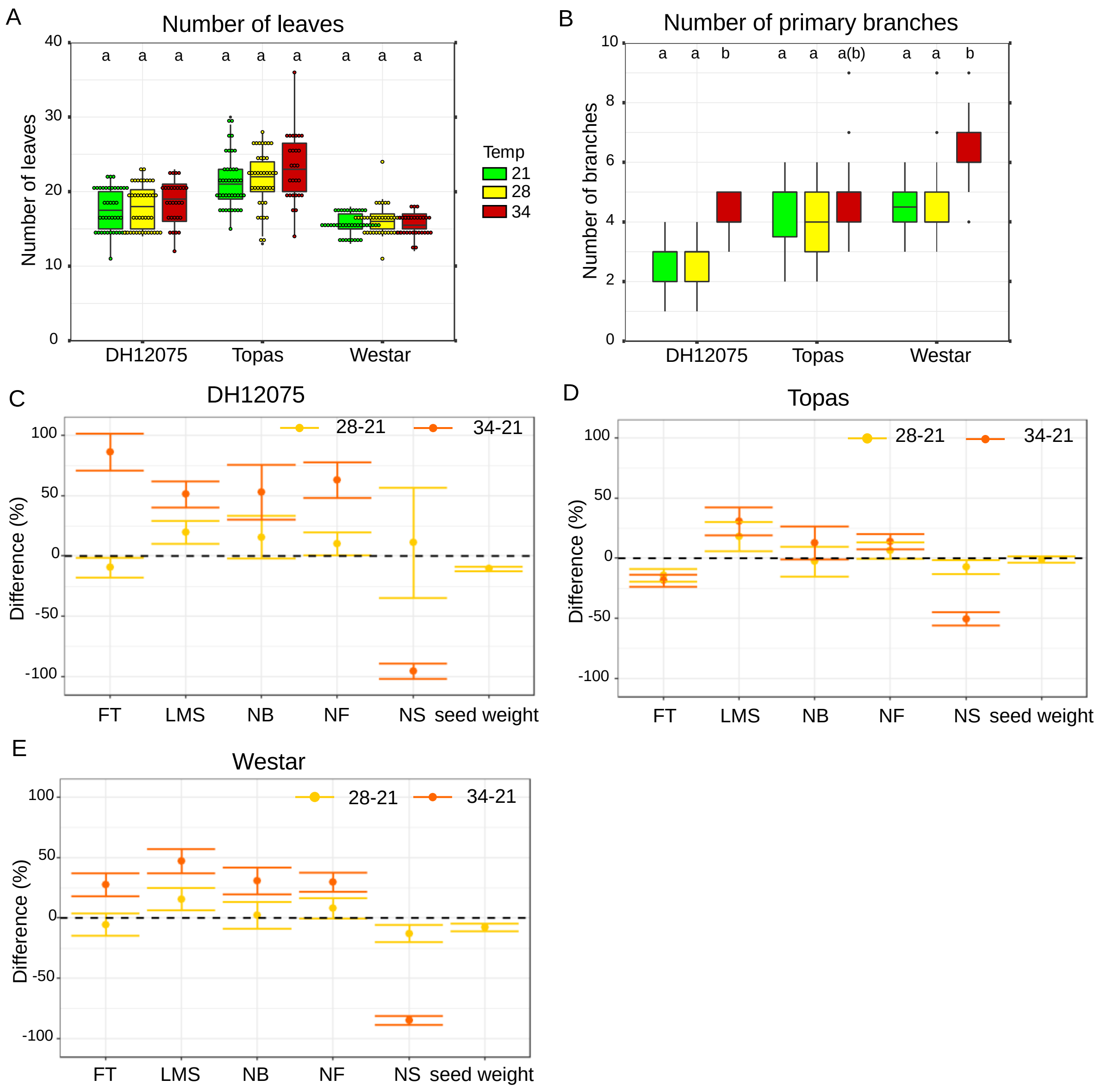

Supplementary Figure 2. High temperatures affect growth parameters of Brassica flowering plants. **(A, B)** The number of leaves(**A**), and the number of primary branches (NB) (**B**) were quantified in DH12075, Topas, and Westar cultivars at CT (21/18 ^{o}C, green), MT (28/18 ^{o}C, yellow), and HT (34/18 ^{o}C, red). Growth parameters are presented as boxplots (the box represents the interquartile range and the line inside the box represents the median). Each dot is an observation**(A)**, or only outliers **(B)**. The Pearson correlation coefficient between LSM, FT and NF is shown in **Supplementary Table S2**. Boxes with the same letters (a, b) within each cultivar do not differ significantly ($p < 0.05$). **(C-E)** Graphs display the differences between CT and MT (yellow), and CT and HT (orange) in flowering time (FT, days), length of the main stem (LMS, cm), number of branches (NB), number of flowers (NF), number of seeds (NS) and seed weight in DH12075 (D), Topas (E) and Westar (F). Dots represent

the mean values of the differences and error bars, the 95 % confidence interval. Data on FT, LMS and NF are presented in Figure 1. Data on NS and seed weight are presented in Figure 5.

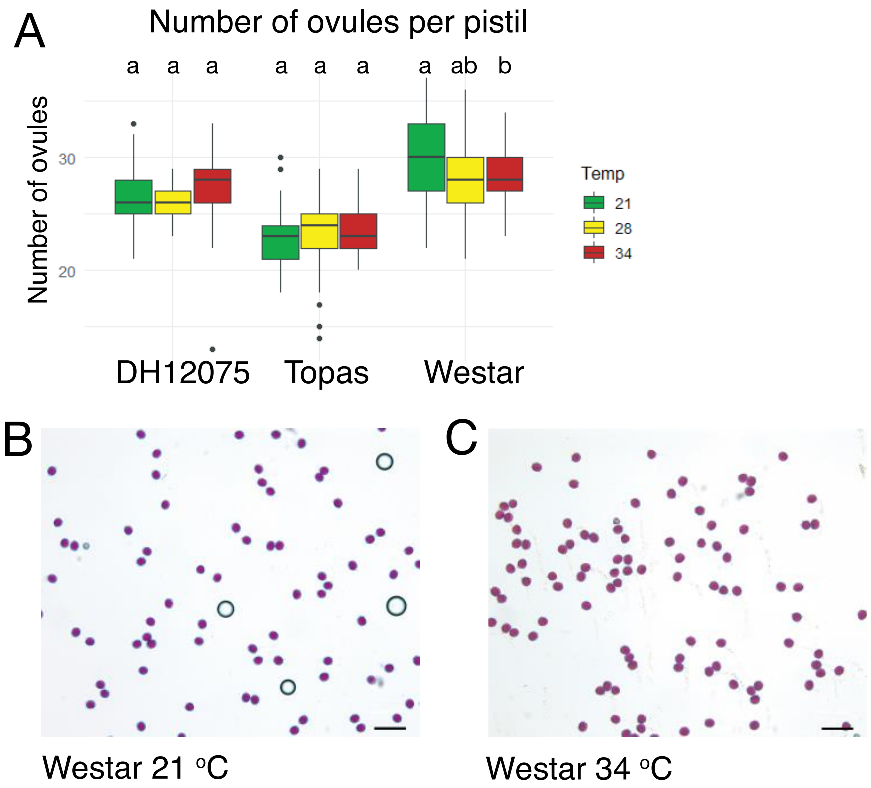

D

	conditions	not viable (%)	n pollen
DH12075	CT	2.23 - 3.65	2294
	MT	2.88 - 4.75	1687
	HT	1.09 - 2.22	2265
Topas	CT	0.36 - 1.30	1510
	MT	2.49 - 5.19	848
	HT	2.85 - 4.86	1489
Westar	CT	0.48 - 1.53	1452
	MT	1.52 - 4.26	605
	HT	1.37 - 3.22	1065

Supplementary Figure S3. The number of ovules and the pollen development are not affected by high temperatures. **(A)** Graph displaying the number of ovules per pistil in DH12075, Topas and Westar cultivars at CT (21/18 °C, green), MT (28/18 °C, yellow) and HT (34/18 °C, red). Relates to Table 1. The number of ovules per pistil is presented as boxplots (the box represents the interquartile range and the line inside the box represents the median). Each dot indicates outliers. Boxes with the same letters (a, b) within each cultivar do no differ significantly ($p < 0.05$). **(B, C)** Pollen grain development is not affected by our growth conditions. Pollen grains of Westar were assayed for viability with Alexander staining. No differences were observed in pollen viability from plants grown at 21 °C **(B)** and 34 °C

(C). Scale bars represent 100 μm. (**D**) Table with the count of viable pollen grains by growth conditions per cultivar. The 95% confidence interval is given as a percentage.

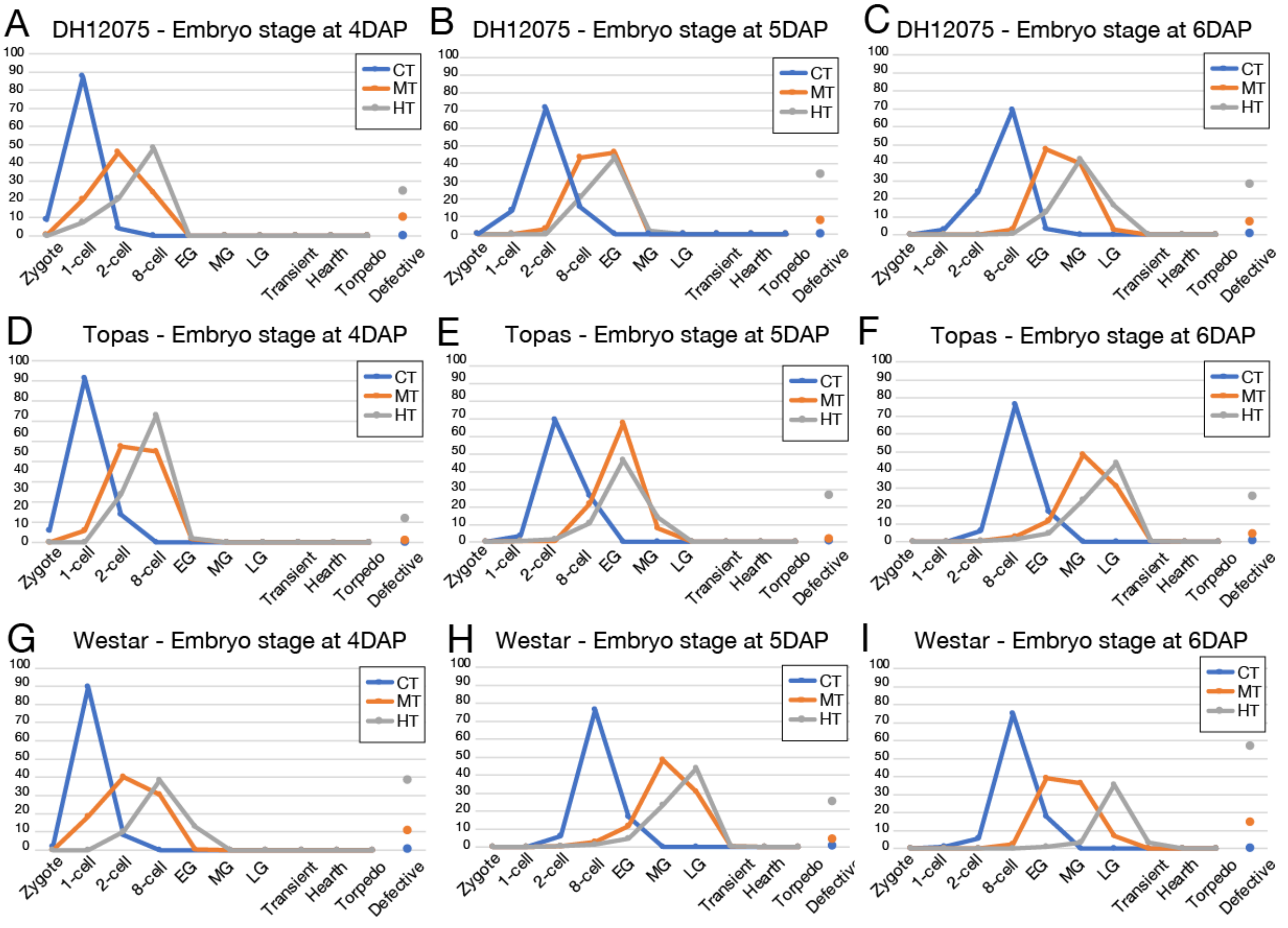

Supplementary Figure S4. Embryo development is accelerated by high temperatures. Graphs displaying the distribution (as a percentage) of embryonic development stages per silique at 4 DAP **(A, D, G)**, 5 DAP **(B, E, H)** and 6 DAP **(C, F, I)** in DH12075 **(A-C)**, Topas **(D-F)**, and Westar plants **(G-I)** grown at CT (21/18 ºC, blue), MT (28/18 ºC, orange) and HT (34/18 ºC, grey). EG, early globular; MG, mid-globular; LG, late globular embryos. Relates to Table 2.

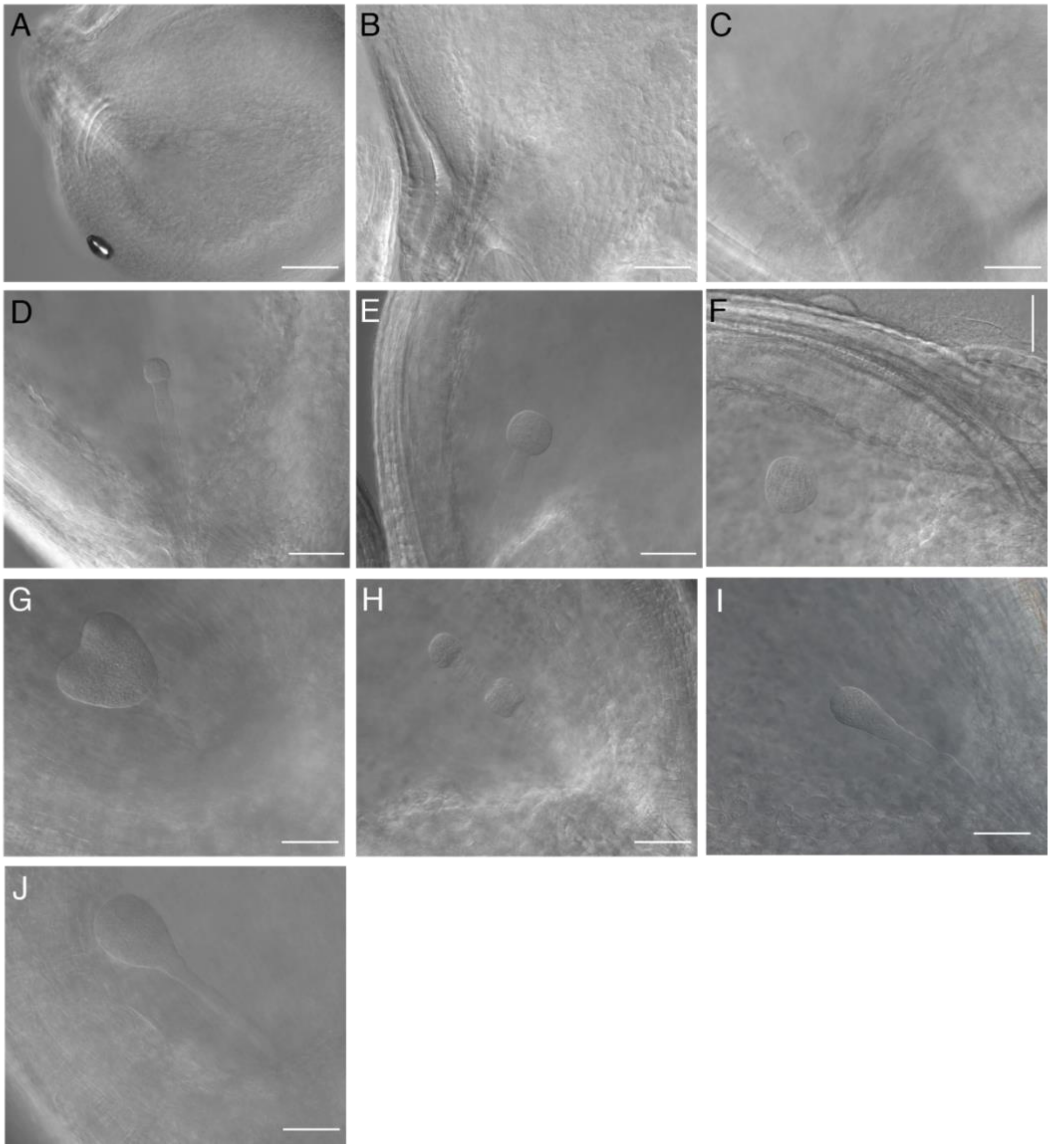

Supplementary Figure S5. High temperatures affect embryo development. Original pictures are presented in Figure 4. (**A-G**) Embryos from plants grown at CT (21/18 ºC). (**H-J**) Range of defective embryos observed in *B. napus* plants grown at MT (28/18 ºC) and HT (34/18 ºC) between 6 and 8 DAP. Scale bars represent 100 µm.

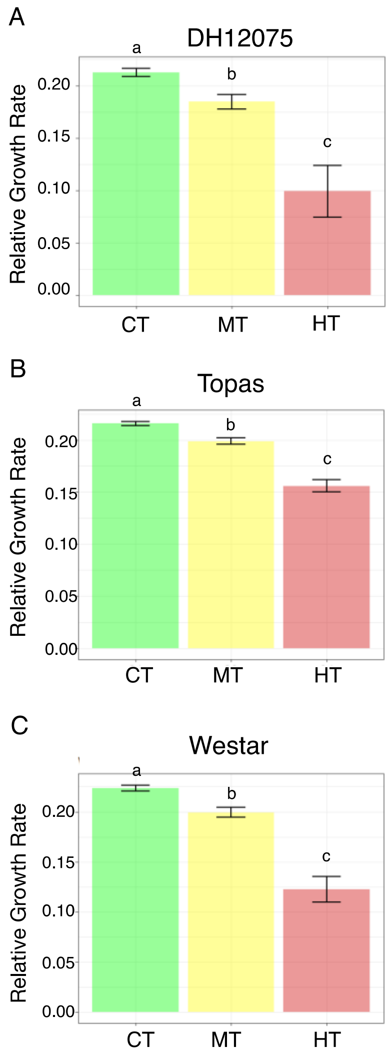

Supplementary Figure S6. Growth rate of siliques is reduced by growth at elevated temperatures. Graphs displaying the relative growth rate of siliques between 0 DAP to 11 DAP in DH12075 (**A**), Topas (**B**) and Westar (**C**) plants grown at CT (green), MT (yellow) and HT (red). Shown are barplots with 95 % confidence intervals. Bars with the different letters (a, b, c) within each cultivar differ significantly ($p < 0.05$).

1.2 Supplementary Tables

Supplementary Table S1. Primers used in qPCR analysis and LOC number of amplified genes

Primer name	Primer sequence	Targeted LOCs in *B. napus*	Homologous genes in *Arabidopsis*
BnaMYB34-FW<br>BnaMYB34 REV	ACGTCGATTCTCCGACCAA<br>TATGAAACCGCTTGACGCTG	LOC106374996<br>LOC106434612	*MYB34*<br>*At5g60890*
BnaCYP79B-FW<br>BnaCYP79B-REV	CTTGGAAGTTGCCTGAGAATG<br>GAACGATGCTCTGGGAGTC	LOC106439341	*CYP79B2*<br>*At4g39950*<br>*CYP79B3*<br>*At2g22330*
BnaDAOs-FW<br>BnaDAOs-REV	TTTGTGGATGCTGAACATCCG<br>TAAGCTTGAGAGCTTCTCCATC	LOC106414697<br>LOC106430970<br>LOC106369131<br>LOC106399859<br>LOC106380301<br>LOC106382334<br>LOC106414671<br>LOC106369133<br>LOC106362101<br>LOC106379949	*DAO1*<br>*At1g14130*<br>*DAO2*<br>*At1g14120*
BnaGH3.9s-FW<br>BnaGH3.9s-REV	ACGTGGTTCTAAGCATCGAC<br>GCGAGGAATGCCTTGTTTTC	LOC106392343<br>LOC106396677<br>LOC106392367<br>LOC106450413	*GH3.9*<br>*At2g47750*
BnaACGH3.1-FW<br>BnaACGH3.1-REV	AGTTGTTAGTGAGGGACGGA<br>CGGTAAACCGAGTTCAACGA	LOC106409452<br>LOC106432954	*GH3.1*<br>*At2g14960*
BnaGH3.5s_FW<br>BnaGH3.5s_REV	AAGAACGCAATGACACACCT<br>TGGCCAGGGATAGAACTTGT	LOC106409348<br>LOC106376087<br>LOC106440950<br>LOC106445777	*GH3.5*<br>*At4g27260*
BnaNCED9-FW2<br>BnaNCED9-REV2	GTGACGGTAAGTTCGGAGGA<br>GATCTCACATTTTCCTCGTCGT	LOC106354557<br>LOC106354700<br>LOC106441091	*NCED9*<br>*At1g78390*
BnaABA1-2 FW1<br>BnaABA1-2 REV1	TTCGGACAAGAAGGCGG<br>AATTCACCGTCGGTTTACTCAA	LOC106390346	*ABA1/ZEP*<br>*At5g67030*
BnaPHYA-FW1<br>BnaPHYA-REV1	TTGTGTCAGAAGCAGCTCAG<br>TCCAGATCCAAGCACCCTTC	LOC106400740<br>LOC106346267<br>LOC106417700<br>LOC106420085	*PHYA*<br>*At1g09570*
BnaELF4-FW1<br>BnaELF4-REV1	GGAGCAGGGAGGAGAAGATC<br>GGTGATTGTCGTTGACCTGC	LOC106420605<br>LOC106394477<br>LOC106394476<br>LOC106438048<br>LOC106399222<br>LOC106454592<br>LOC106399325	*ELF4*<br>*At2g40080*

BnaFT-FW2<br>BnaFT-REV2	GAGGTGACAAATGGGTTGGA<br>GCTAGGACTTGGAACATCTGG	LOC106402982<br>LOC111213971<br>LOC111207300<br>LOC106426212	*FT*<br>*At1g65480*<br>*TSF*<br>*At4g20370*
BnaRNPII37c-FW1<br>BnaRNPII37c-REV1	TAACGACAAGGGAAGGCTGT<br>GTGTTCCTCATCCTCTGCCT	LOC106377472<br>LOC106353095	*HSP70*<br>*At3g12580*
BnaSCL30A-FW2<br>BnaSCL30A-REV2	AACGATCAAGGGGAAGGACA<br>CCTGTAGACTGGCGAACGT	LOC106412002<br>LOC106452514	*SCL30A*<br>*At3g13570*
BnaSLU7-FW1<br>BnaSLU7-REV1	GTCGTGGTGGAAGGATCAAC<br>CTTCCTCAGCCGCCTCTATC	LOC106446239<br>LOC106453449	*SMP1*<br>*At1g65660*
BnaFBA6-FW2<br>BnaFBA6-REV2	GTTGAAGACTTGGGGAGGGA<br>TCAGAGTTAGCCTTGCACCT	LOC106391482<br>LOC106395718<br>LOC106367801<br>LOC111206734<br>LOC106367922<br>LOC106452433<br>LOC106428297<br>LOC106445170	*FBA6*<br>*At2g36460*
BnaDRM2-FW2<br>BnaDRM2-REV2	TCTCACCCAAACTCTCCCAC<br>TCACCAGCATGTCACTCCAT	LOC106451187<br>LOC106347199<br>LOC106437808	*DRM2*<br>*At2g33830*
BnaTMA7-FW1<br>BnaTMA7-REV1	TTCCTGTGTTTTATCCATGTAGCC<br>CAGTCACTCTCCTACGAACATGATAG	LOC106452133	*TMA7*<br>*At1g15270*<br>*At3g16040*
BnaACT7-FW1<br>BnaACT7-REV1	GAGCAGCATGAAGATCAAGGT<br>CTTCGAGATCCACATCTGTTGG	LOC106382989<br>LOC106426760<br>LOC106426759	*ACT7*<br>*At5g09810*
BnaeIF5A-FW1<br>BnaeIF5A-REV1	ATCTCAGCGCTCTGATGAAGA<br>ACTATTGGTTTACTTGCCACC	LOC106389545	*eIF5A*<br>*At1g13950*<br>*At1g26630*<br>*At1g69410*

Supplementary Table S2. Pearson correlation coefficient between the length of the main inflorescence stem, the flowering time duration and the number of flowers.

	LMSxFT			LMSxNF			FTxNF		
	21	28	34	21	28	34	21	28	34
DH12075	0.88	0.63	0.78	0.77	0.43	0.77	0.76	0.57	0.23
Topas	0.80	0.79	0.41	0.81	0.90	0.44	0.79	0.77	0.37
Westar	0.81	0.63	0.60	0.81	0.74	0.22	0.76	0.58	0.22

Strong correlation 1-0.5, medium 0.5 -0.3, small < 0.3

Supplementary Table S3. Auxin and auxin metabolites measurements (source data of Figures 2 and 7). One representative biological replicate is presented.

sample		average		S.D.	av.		S.D.	av.		S.D.
**type**	**temp**	**TRP**			**IAOx**			**ANT**		
**Pistil**	**21 CT**	26 006.7	±	5 356.7	194.9	±	42.5	408.5	±	113.7
		RSD		21%	RSD		22%	RSD		28%
	**34 HT**	43 330.1	±	4 248.1	155.8	±	23.8	436.4	±	160.6
		RSD		10%	RSD		15%	RSD		37%
		**	/	0.00203	-	/	0.18946	-	/	0.78438
**5 DAP**	**21 CT**	79 755.2	±	18 901.1	34.4	±	2.5	410.9	±	193.1
		RSD		24%	RSD		7%	RSD		47%
	**34 HT**	31 326.1	±	8 433.4	<LOD			357.4	±	154.2
		RSD		27%				RSD		43%
		**	/	0.00158				-	/	0.69579
**26 DAP**	**21 CT**	9 491.1	±	7 234.0	14 728.9	±	1 560.1	*NQ*		
		RSD		76%	RSD		11%			
	**34 HT**	26 807.7	±	11 659.5	6 946.5	±	541.2	*NQ*		
		RSD		43%	RSD		8%			
		*	/	0.03558	***	/	0.00007			

sample		average		S.D.	av.		S.D.	av.		S.D.
**type**	**temp**	**IPyA**			**IAM**			**IAN**		
**Pistil**	**21 CT**	12 271.6	±	2 001.4	1 123.2	±	241.9	92 234.3	±	4 992.8
		RSD		16%	RSD		22%	RSD		5%
	**34 HT**	15 655.5	±	3 640.1	2 550.0	±	766.7	217 595.1	±	14 508.7
		RSD		23%	RSD		30%	RSD		7%
		-	/	0.14192	*	/	0.01595	***	/	0.00000
**5 DAP**	**21 CT**	7 435.3	±	802.1	270.6	±	15.3	17 592.6	±	1 011.9
		RSD		11%	RSD		6%	RSD		6%
	**34 HT**	6 755.5	±	961.2	260.6	±	53.3	28 585.4	±	2 034.0
		RSD		14%	RSD		20%	RSD		7%
		-	/	0.30912	-	/	0.75714	***	/	0.00005
**26 DAP**	**21 CT**	14 813.1	±	2 759.9	*NQ*			33 648.6	±	2 111.6
		RSD		19%				RSD		6%
	**34 HT**	7 990.2	±	992.5	*NQ*			12 648.1	±	984.5
		RSD		12%				RSD		8%
		**	/	0.00164				***	/	0.00000

sample		average		S.D.	av.		S.D.	av.		S.D.
**type**	**temp**	**IAA**			**oxIAA**			**IAAsp**		
**Pistil**	**21 CT**	429.7	±	171.1	292.9	±	43.4	6.6	±	0.8
		RSD		40%	RSD		15%	RSD		12%
	**34 HT**	311.0	±	11.2	298.9	±	18.4	4.5	±	1.3
		RSD		4%	RSD		6%	RSD		29%
		-	/	0.20350	-	/	0.81269	-	/	0.05361
**5 DAP**	**21 CT**	2 746.6	±	108.8	11 409.7	±	603.0	43.8	±	2.4
		RSD		4%	RSD		5%	RSD		6%
	**34 HT**	2 132.2	±	83.1	15 045.6	±	914.7	23.1	±	2.1
		RSD		4%	RSD		6%	RSD		9%
		***	/	0.00006	***	/	0.00052	***	/	0.00001
**26 DAP**	**21 CT**	14 152.9	±	371.3	110 339.3	±	6 979.5	87.5	±	6.1
		RSD		3%	RSD		6%	RSD		7%
	**34 HT**	3 904.8	±	585.7	44 577.9	±	3 869.0	2 087.7	±	172.8
		RSD		15%	RSD		9%	RSD		8%
		***	/	0.00000	***	/	0.00000	***	/	0.00000

sample		average		S.D.	av.		S.D.	av.		S.D.
**type**	**temp**	**IAGlu**			**IAA-Glc**			**oxIAA-Glc**		
**Pistil**	**21 CT**	*<LOD*			143.3	±	29.7	2 923.0	±	169.6
					RSD		21%	RSD		6%
	**34 HT**	*<LOD*			150.8	±	18.2	1 690.0	±	83.5
					RSD		12%	RSD		5%
					-	/	0.69455	***	/	0.00000
**5 DAP**	**21 CT**	62.9	±	6.4	876.5	±	57.3	43 524.6	±	2 863.5
		RSD		10%	RSD		7%	RSD		7%
	**34 HT**	54.4	±	9.0	478.4	±	68.5	43 785.4	±	1 864.8
		RSD		17%	RSD		14%	RSD		4%
		-	/	0.20301	***	/	0.00008	-	/	0.88867
**26 DAP**	**21 CT**	17.2	±	1.8	*NQ*			788 378.5	±	46 734.7
		RSD		11%				RSD		6%
	**34 HT**	96.1	±	5.8	*NQ*			150 035.7	±	11 672.8
		RSD		6%				RSD		8%
		***	/	0.00000				***	/	0.00000

NQ: not quantified; <LOD: below the limit of detection; CT: control temperature; HT: hight temperature; av.: average; S.D.; standard deviation; RSD: relative standard deviation (ratio of the standard deviation over the average). Asterisks indicate statistically significant difference in HT in a paired Student's t-test (t-test; *, **, and *** correspond to P-values of 0.05 > p > 0.01, 0.01 > p > 0.001, and p < 0.001, respectively). All measurements are as pmol/g FW.

Supplementary Table S4. ABA measurements (source data of Figures 3 and 6)

sample				
type	*temp.*	average		S.D.
		**ABA**		
**Pistil**	**21 CT**	1 101.6	±	77.8
		RSD		7%
	**34 HT**	513.6	±	30.3
		RSD		6%
		***	/	0.00000
**5 DAP**	**21 CT**	1 329.7	±	32.0
		RSD		2%
	**34 HT**	1 372.4	±	63.8
		RSD		5%
		-	/	0.31725
**26 DAP**	**21 CT**	6 750.6	±	283.6
		RSD		4%
	**34 HT**	2 567.6	±	100.3
		RSD		4%
		***	/	0.00000

NQ: not quantified; CT: control temperature; HT: high temperature; S.D.; standard deviation; RSD: relative standard deviation (ratio of the standard deviation over the average). Asterisks indicate a statistically significant difference in HT in a paired Student's t-test (t-test; *** correspond to P-values of $p < 0.001$).

Supplementary Table S5. Pearson correlation coefficient between the siliques growth rate, the seed number per silique and the growth temperatures

Cultivars	CT (21ºC)	MT (28ºC)	HT (34ºC)
DH12075	0.83	0.96	0.99
Topas	0.63	0.92	0.92
Westar	0.67	0.92	0.96

Strong correlation 1-0.5, medium 0.5 -0.3, small < 0.3

Supplementary Table S6. Glucosinolates, Nitrogen and seed oil measurement (source data of Figure 9).

sample		av.		S.D.	av.		S.D.	av.		S.D.
**Cult.**	**temp**	**GSL in 9% humidity**			**Nitrogen compound**			**Oil in dry matter**		
**Topas**	**21**	10.61	±	0.46	21.11	±	0.63	42.33	±	1.55
	**CT**	RSD		4.36%	RSD		2.97%	RSD		3.65%
	**28**	12.77	±	2.05	26.71	±	0.70	40.14	±	1.73
	**MT**	RSD		16.05%	RSD		2.61%	RSD		4.30%
		*	/	0.01008	***	/	0.00000	*	/	0.01664
**Westar**	**21**	8.61	±	1.07	20.94	±	0.52	43.94	±	1.20
	**CT**	RSD		12.38%	RSD		2.48%	RSD		2.74%
	**28**	11.05	±	1.87	24.78	±	0.65	39.00	±	0.83
	**MT**	RSD		16.89%	RSD		2.64%	RSD		2.12%
		**	/	0.00540	***	/	0.00000	***	/	0.00000
**DH12075**	**21**	11.51	±	0.79	20.30	±	0.72	42.27	±	1.97
	**CT**	RSD		6.91%	RSD		3.54%	RSD		4.66%
	**28**	16.80	±	1.35	24.12	±	0.99	36.24	±	1.49
	**MT**	RSD		8.02%	RSD		4.12%	RSD		4.11%
		***	/	0.00000	***	/	0.00000	***	/	0.00000

sample		av.		S.D.	av.		S.D.		
**Cult.**	**temp**	**Palmitic acid**			**Stearic acid**			**ODP**	**LDP**
**Topas**	**21**	4.23	±	0.17	2.40	±	0.19	27.3895	31.2718
	**CT**	RSD		4.04%	RSD		7.99%		
	**28**	3.71	±	0.17	2.50	±	0.28		
	**MT**	RSD		4.60%	RSD		11.29%	24.9661	27.8822
		***	/	0.00002	-	/	0.42912		
**Westar**	**21**	4.03	±	0.16	2.30	±	0.12	23.1731	28.9365
	**CT**	RSD		3.90%	RSD		5.12%		
	**28**	3.90	±	0.16	2.21	±	0.16		
	**MT**	RSD		4.19%	RSD		7.36%	20.3773	25.7554
		-	/	0.11280	-	/	0.20737		
**DH12075**	**21**	4.09	±	0.16	2.21	±	0.21	25.0497	31.1685
	**CT**	RSD		4.02%	RSD		9.68%		
	**28**	4.20	±	0.12	2.55	±	0.12		
	**MT**	RSD		2.86%	RSD		4.76%	22.1162	26.4949
		-	/	0.17945	**	/	0.00129		

sample		av.		S.D.	av.		S.D.	av.		S.D.
**Cult.**	**temp**	**Oleic acid**			**Linoleic acid**			**Linolenic acid**		
**Topas**	**21**	66.63	±	0.56	17.27	±	0.63	7.86	±	0.30
	**CT**	RSD		0.84%	RSD		3.63%	RSD		3.79%
	**28**	69.49	±	1.29	16.67	±	0.90	6.45	±	0.33
	**MT**	RSD		1.86%	RSD		5.42%	RSD		5.11%
		***	/	0.00003	-	/	0.14231	***	/	0.00000
**Westar**	**21**	70.63	±	0.96	15.14	±	0.77	6.16	±	0.33
	**CT**	RSD		1.36%	RSD		5.08%	RSD		5.39%
	**28**	74.00	±	0.66	14.06	±	0.70	4.88	±	0.22
	**MT**	RSD		0.89%	RSD		5.00%	RSD		4.53%
		***	/	0.00000	**	/	0.00990	***	/	0.00000
**DH12075**	**21**	68.34	±	1.16	15.72	±	0.80	7.12	±	0.37
	**CT**	RSD		1.70%	RSD		5.10%	RSD		5.26%
	**28**	70.93	±	1.09	14.81	±	1.15	5.34	±	0.30
	**MT**	RSD		1.53%	RSD		7.76%	RSD		5.60%
		***	/	0.00029	-	/	0.08300	***	/	0.00000

CT: control temperature; MT: mid temperature; av.: average; S.D.; standard deviation; RSD: relative standard deviation (ratio of the standard deviation over the average); ODP: Oleic desaturation proportion; LDP: Linoleic desaturation proportion. Asterisks indicate statistically significant difference in MT compared to CT in a paired Student's t-test (t-test; *, **, and *** correspond to P-values of $0.05 > p > 0.01$, $0.01 > p > 0.001$, and $p < 0.001$, respectively). GSL is quantified as µmol/g FW. Nitrogen compound and total oil in dry matter are quantified as % of FW. Fatty acids are quantified as % of total oil content.
